# Kidney tubular epithelial cells control interstitial fibroblast fate by releasing TNFAIP8-encapsulated exosomes

**DOI:** 10.1038/s41419-023-06209-w

**Published:** 2023-10-12

**Authors:** Xi Liu, Zhao Liu, Cong Wang, Jinhua Miao, Shan Zhou, Qian Ren, Nan Jia, Lili Zhou, Youhua Liu

**Affiliations:** 1 State Key Laboratory of Organ Failure Research, National Clinical Research Center of Kidney Disease, Division of Nephrology, Nanfang Hospital, Southern Medical University, Guangzhou, China; 2 Guangdong Provincial Institute of Nephrology, Guangzhou, China

**Keywords:** Kidney diseases, Cell biology

## Abstract

Kidney fibrosis, characterized by the activation and expansion of the matrix-producing fibroblasts, is the common outcome of chronic kidney disease (CKD). While fibroblast proliferation is well studied in CKD, little is known about the regulation and mechanism of fibroblast depletion. Here, we show that exosomes derived from stressed/injured tubules play a pivotal role in dictating fibroblast apoptosis and fate. When human kidney tubular cells (HK-2) were stimulated with TGF-β1, they produced and released increased amounts of exosomes (TGFβ-Exo), which prevented renal interstitial fibroblasts from apoptosis. In vivo, injections of TGFβ-Exo promoted renal fibroblast survival, whereas blockade of exosome secretion accelerated fibroblast apoptosis in obstructive nephropathy. Proteomics profiling identified the tumor necrosis factor-α-induced protein 8 (TNFAIP8) as a key component enriched in TGFβ-Exo. TNFAIP8 was induced in renal tubular epithelium and enriched in the exosomes from fibrotic kidneys. Knockdown of TNFAIP8 in tubular cells abolished the ability of TGFβ-Exo to prevent fibroblast apoptosis. In vivo, gain- or loss- of TNFAIP8 prevented or aggravated renal fibroblast apoptosis after obstructive injury. Mechanistically, exosomal-TNFAIP8 promoted p53 ubiquitination leading to its degradation, thereby inhibiting fibroblasts apoptosis and inducing their proliferation. Collectively, these results indicate that tubule-derived exosomes play a critical role in controlling the size of fibroblast population during renal fibrogenesis through shuttling TNFAIP8 to block p53 signaling. Strategies to target exosomes may be effective strategies for the therapy of fibrotic CKD.

## Introduction

Chronic kidney disease (CKD) has become a public health problem worldwide, with the prevalence ranging from 10 to 15% in the adult population [1–3]. Irrespective of the initial causes, kidney fibrosis is considered as the ultimate outcome of virtually all kinds of CKD [4, 5]. In various pathologic conditions, tubular epithelium is the epicenter and primary target of kidney injury [6]. The stressed/injured tubular cells undergo a variety of intracellular changes and mediate a series of intercellular communications by releasing soluble factors and extracellular vesicles (EVs) such as exosomes, which result in activation of the interstitial fibroblasts characterized by cell proliferation and excessive production of extracellular matrix (ECM) leading to tissue scarring [7–9]. As the principal matrix-producing cells, the pool size of activated fibroblasts population could be a major determinant that dictates the prognosis of kidney fibrosis. While fibroblast activation and proliferation in CKD are extensively studied [10], the relative role of fibroblast depletion via apoptosis in governing kidney fibrosis remains largely unexplored.

Exosomes are small, lipid bilayer membranous vesicles with a size range of 40–160 nm in diameter that are generated from intracellular multivesicular bodies (MVBs) [11–13]. Diverse types of cells under different physiologic and pathologic conditions can produce and secret exosomes. In diseased kidney, the majority of exosomes are originated from stressed tubular cells and glomerular podocytes. The components of exosomes include an assortment of bioactive molecules, such as membrane proteins, growth factors and cytokines, ECM proteins, lipids, mRNAs, microRNA, and long noncoding RNAs [13–16]. By shuttling these materials and signals, exosomes play an imperative role in mediating cell-cell communication and modulating the behavior of the recipient cells. Previous studies from our group and others have shown that exosomes derived from injured tubular cells are capable of transferring sonic hedgehog (Shh) protein and TGF-β1 mRNA into fibroblasts, stimulating their activation and proliferation in the fibrotic kidneys [17, 18]. However, whether tubule-derived exosomes play a role in regulating fibroblast apoptosis and depletion in CKD is completely unknown.

In the present study, we demonstrate that tubule-derived exosomes prevented renal interstitial fibroblasts from apoptosis, leading to an expanded fibroblast population in CKD. Through proteomic profiling, we identified tumor necrosis factor-α-induced protein 8 (TNFAIP8) encapsulated in tubule-originated exosomes as a key mediator. Our findings highlight the importance of tubule-derived, exosomal-TNFAIP8 in controlling the fate and population size of interstitial fibroblasts in the pathogenesis of CKD.

## Materials and methods

### Cell culture and treatment

Human proximal tubular epithelial cells (HK-2) and normal rat kidney interstitial fibroblast cells (NRK-49F) were obtained from the American Type Culture Collection (Manassas, VA). After serum starvation overnight, HK-2 cells were treated with 2 ng/ml recombinant human TGF-β1 (R & D Systems, Minneapolis, MN) for 6 h and then TGF-β1 was removed, followed by incubating in serum-free medium for 48 h. In some experiments, HK-2 cells were pretreated with 100 µg/ml dimethyl amiloride (DMA) (Sigma) or transfected with TNFAIP8 siRNA using Lipofectiamine 2000 (Life Technologies, Grand Island, NY). The conditioned media were collected and subjected to exosomes isolation. Following serum starvation for 16 h, NRK-49F cells were treated with HK-2 cell conditioned media or exosomes (30 µg) isolated from HK-2 cell conditioned media. For some experiments, NRK-49F cells were pretreated with cisplatin 20 µM (Sigma, St. Louis, MO) to induce cell apoptosis, followed by incubation with 10 µM p53 inhibitor Pifithrin-α (Selleck Chemicals) or MG132 (Selleck Chemicals), respectively. Cells after different treatments were collected and subjected to various analyses.

### Animal model

Male C57BL/6 mice, weighing approximately 20–22 g, were purchased from Vital River Laboratory (Beijing, China) and housed in the standard environment with regular light/dark cycles and free access to water and chow diet. Mice were randomized into different groups using the online tool “Research Randomizer” (https://www.randomizer.org). Six mice were included in each group to meet the minimum sample size requirement to perform an Analysis of Variance (ANOVA) test. For unilateral ureteral obstruction (UUO) model, mice were subjected to double-ligating the left ureter using 4–0 silk after a midline abdominal incision. Sham operated mice had their ureters exposed, manipulated but not ligated. Mice were sacrificed at 14 days after UUO. Renal unilateral ischemia-reperfusion injury (UIRI) was established as described [3, 19]. Briefly, left renal pedicles were clipped for 35 min using microaneurysm clamps for IRI. During the ischemic period, body temperature was maintained between 37–38 °C using a temperature-controlled heating system. After removal of the clamps, reperfusion of the kidneys was visually confirmed. The intact right kidney was removed via a right flank incision at 10 days post-UIRI, and mice were killed a day thereafter. The kidney tissues were collected for various analyses.

For studying the effects of exosomes, five sets of experiments were performed, and the experimental design was detailed in corresponding figures. Exogenous exosomes collected from HK-2 cells treated with or without TGF-β1 were quantified by using the micro bicinchoninic acid (BCA) protein assay and injected intravenously via tail vein (200 μg per mouse per time point). For inhibiting exosomes generation in vivo, daily intraperitoneal injections of DMA at the dose of 20 mg/kg body weight in 0.9% saline were performed. For some experiments, mice were administered by rapid injection of pLVX-shRab27a, pFlag-TNFAIP8, pLVX-shTNFAIP8 expression plasmids or empty vector pcDNA3 through the tail vein, as described previously [17, 20]. The animal studies were conducted according to NIH Guide for the Care and Use of Laboratory Animals and approved by the Animal Experimentation Committee at the Nanfang Hospital, Southern Medical University.

### Determination of serum creatinine and blood urea nitrogen

Serum creatinine and blood urea nitrogen (BUN) levels were determined by an automatic chemistry analyzer (AU480; Beckman coulter, Pasadena, California). The levels of serum creatinine and BUN were expressed as mg/dl.

### Exosomes isolation

Exosomes were isolated from conditioned media by differential centrifugation. Briefly, after removing cells and other debris by centrifugation at 300 × *g* for 10 min, 2000 × *g* for 20 min, and 10,000 × *g* for 30 min, respectively, the supernatant was ultra-centrifuged at 110,000 × *g* for 1 h, and all steps were performed at 4 °C. The pellets were suspended in ice-cold phosphate-buffered saline (PBS) to remove contaminating protein, then collected by ultra-centrifuging at 110,000 × *g* for 1 h. The final pellets of exosomes were resuspended in serum-free culture medium for treatment with NRK-49F cells for 24 h or in ice-cold PBS for tail vein injection to mice. Some exosomes were also used for Western blot analyses.

### Nanoparticle tracking analysis (NTA)

Freshly concentrated exosome solution was diluted in PBS (1:1000) and gently pipetted before injected into a Nanosight NTA NS300 instrument (Malvern Instruments Inc., Westborough, MA) using a sterile injection syringe. The dynamic images were recorded and analyzed to obtain the concentration and distribution of the particles.

### Transmission electron microscopy (TEM)

For transmission electron microscopy (TEM), the pelleted exosomes isolated from TGF-β1-treated HK-2 cells or small pieces of kidney tissue from IRI mice were placed in 2.5% glutaraldehyde in PBS buffer and fixed. Samples were rinsed and post-fixed in 1% osmium tetroxide, then dehydrated in increasing concentrations of alcohol and infiltrated with increasing concentrations of epoxy resin mixed with propylene oxide. The samples were embedded in resin and cut into ultrathin sections with a diamond knife. The sections were then placed on copper mesh grids and stained with the heavy metal uranyl acetate for contrast. Samples were observed by a Tecnai transmission electron microscope at 120 kV (Thermo Fisher Scientific, Hillsboro, OR).

### 4D-Label free proteomics analysis

The primary experimental procedures for 4D-Label free proteomics analysis include protein extraction, trypsin digestion, LCMS/MS analysis and data analysis. The resulting MS/MS data were processed using the MaxQuant search engine (v.1.6.15.0). Tandem mass spectra were searched against the Human UniProt database concatenated with a reverse decoy database. GO enrichment analysis of the differentially expressed protein in exosomes from HK-2 cells with or without TGF-β1-treated was identified by the clusterProfiler R package. The proteomics analysis in our studies was supported by Aksomics (Shanghai, China).

### Fluorescent labeling of exosomes

HK-2 cells were labeled with cell tracker Dil, a fluorescent lipophilic cationic indocarbocyanine dye (Life Technologies, Grand Island, NY) for 1 h and then washed three times with PBS. After TGF-β1 treatment, exosomes were harvested from the conditioned media of Dil-labeled HK-2 cells. EVs were resuspended, and then incubated with NRK-49F cells for 24 h or injected into mice via the tail vein, and then detected by immunofluorescence.

### Treatment with exosomes in vitro and in vivo

The exosome content from conditioned media was analyzed using the microbicinchoninic acid assay and the reminiscent was used for treatment after being resuspended in PBS. NRK-49F cells were treated with 30 μg/ml of exosomes for 24 h. For tail vein injection to mice, 200 μg of exosomes was used in one mouse per time point.

### Organ imaging of exosome distribution

C57BL/6 mice were subjected to UUO surgery, following intravenously injection with 200 μg TGFβ-Exo in 400 μl PBS. All mice were sacrificed at 24 h after the final injection. Major organs including kidneys, heart, liver, lung and spleen were removed and placed in glass dishes. Organs were exposed to a Bruker FX PRO imaging system equipped with an excitation at 549 nm and emission at 565 nm, and images were taken with camera and analyzed digitally. All procedures were conducted in dark.

### Primary kidney fibroblasts isolation

Fresh renal tissue (approximately 10 mg) was placed in a sterile Petri dish and soaked with PBS to remove blood. The outer cortex was cut from the medulla to form pieces about 1 mm^3^ and then washed three times. The diced fragments were transferred to a solution of 1 mg/ml collagenase (type IV) in PBS, placed in a water bath at 37 °C for 30 min. The digestion was then stopped by adding an equal volume of ice-cold PBS/10% fetal bovine serum (FBS). The glomeruli and any undigested tubular fragments were removed from the solution by passing through a sterilized 70-mm sieve, the filtrate collected and centrifuged at 4 °C at 200 g for 5 min. The pellets were resuspended in the cold 50% Percoll solution and centrifuged at 30,000 *×* *g* at 4 °C for 30 min. The layer of fibroblasts was harvested, then diluted in ice-cold PBS and centrifuged at 200 × *g* at 4 °C for 5 min to remove any contaminated Percoll. After centrifugation, the supernatant was removed carefully, and the pellets resuspended in culture medium for Western blot analysis or immunofluorescence analyses.

### Western blot analysis

Kidney tissues, cells and exosomes pellets were lysed in radioimmunoprecipitation (RIPA) assay buffer (Beyotime Biotechnology, Shanghai, China) and incubated at 4 °C for 30 min. The protein concentration was measured by BCA protein assay. Equal amounts of protein were separated by 10% sodium dodecyl-sulfate-polyacrylamide gel electrophoresis (SDS-PAGE) and transferred onto polyvinylidene fluoride membranes (Millipore, Darmstadt, Germany). Membranes were blocked with 5% skim milk in Tris-buffered saline with 0.1% Tween 20 (TBST) buffer for 1 h and incubated with primary antibodies overnight at 4 °C. After washing with TBST buffer, membranes were further incubated with appropriate horseradish peroxidase (HRP)-conjugated secondary antibodies at room temperature for 2 h. Protein bands were visualized with the HRP-enhanced chemiluminescence Western blotting substrate. The primary antibodies and secondary antibodies used were listed in Supplementary Table S1.

### RNA extraction and real-time qPCR

Total RNA was isolated and purified from cells and kidney tissue by TRIzol RNA isolation system (Life Technologies, Grand Island, NY), according to the manufacturer’s instruction. RNA concentration was measured by NANODROP ONE (Thermo Fisher Scientific). The first strand of complementary DNA was synthesized using 1 µg of RNA in 20 µl of reaction buffer containing AMV-RT and random primers at 42 °C for 60 min. Quantitative, real-time PCR was performed using a Platinum SYBR Green qPCR SuperMix-UDG kit (Invitrogen). All processes were performed by the manufacturer’s instructions. Relative gene expression (fold induction versus controls) was identified with the 2 − ΔΔCt method. The sequences of the primer pairs were given in Supplementary Table S2.

### Histology and immunohistochemical staining

Formalin-fixed Paraffin-embedded mouse kidney sections (4 µm) were prepared, dewaxed, washed in down-graded alcohols and water by a routine procedure. Hematoxylin-eosin (HE) staining, Masson’s trichrome staining (MTS) and Picrosirius red staining were performed using routine protocol [21]. For immunohistochemical staining, deparaffinized sections were incubated with 3% H_2_O_2_ solution to inhibit endogenous peroxidase activity. After blocking with 10% normal donkey serum, these slides were incubated with primary antibodies and secondary antibodies. Visualization was facilitated by the AEC or DAB substrate solution (Vectorlabs). Haematoxylin was used for nuclear counterstain. The primary antibodies and secondary antibodies used were listed in Supplementary Table S1.

### Immunofluorescence staining

Kidney cryosections were fixed with 3.7% paraformalin for 15 min at room temperature. HK-2 or NRK-49F cells cultured on coverslips were fixed with cold methanol: acetone (1:1) for 15 min at room temperature, followed by blocking with 0.3% Triton X-100 (Sigma-Aldrich) for 15 min and 10% normal donkey serum in PBS for 1 h at room temperature. Slides were then incubated with primary antibodies at 4 °C overnight. After washing, the slides were then incubated with Cy3 or Cy2-conjugated donkey anti-mouse or donkey anti-rabbit IgG (Jackson Immuno-Research Laboratories, West Grove, PA) at room temperature for 2 h. Nuclei were stained with DAPI (Sigma-Aldrich) according to manufacturer’s instruction. The slides were then observed under a confocal microscope (Leica SP8; Leica Microsystems, Buffalo Grove, IL).

### Flow cytometry

For apoptosis assay, isolated NRK-49F cells were stained with Annexin V (Cat. No. 550474, BD Biosciences) and PI (Cat. No. 556547, BD Biosciences) according to the manufacturer’s instructions.

### TUNEL staining

Apoptotic cells were detected using in situ cell death detection kit (Roche-11684795910, Sigma-Aldrich). The slides were also incubated with DAPI (Cat. No. C1005, Beyotime Biotechnology). All procedures were conducted according to the manufacturer’s protocol. Images were taken by fluorescence microscopy (Leica SP8; Leica Microsystems, Buffalo Grove, IL).

### Quantifications of staining

Immunohistochemical, immunofluorescence was quantified at high-powered (×400, ×630) fields from randomly selected 3 fields each section. Quantification of positive staining was assessed by two researchers who were blinded through Image Pro Plus software.

### Statistical analysis

All data examined were expressed as mean ± standard error of mean (SEM). Statistical analyses of the data were performed using GraphPad Prism 7.0/8.0 (GraphPad Software, San Diego, CA). A two-tailed unpaired Student’s *t* test was used for comparison between two groups, and comparisons between multiple groups were made using one-way ANOVA, followed by Student–Newman–Kuels test or Dunnett’s T3 procedure. *P* < 0.05 was considered statistically significant.

## Results

### Tubular cell-derived exosomes protect interstitial fibroblasts from apoptosis

**Fig. 1:**
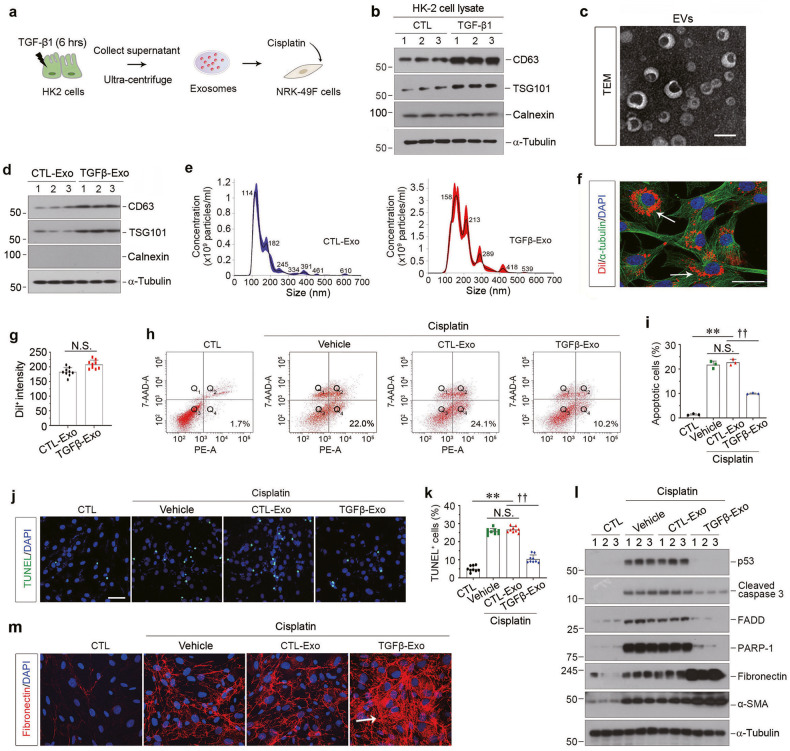
Injured tubular cells-derived exosomes protect fibroblasts from apoptosis induced by cisplatin in vitro. **a** Diagram shows the experimental design. Human kidney proximal tubular epithelial cells (HK-2) were treated with TGF-β1 (2 ng/ml) for 6 h, and then removed TGF-β1 and continued to incubate for additional 48 h in serum-free medium. Exosomes were isolated from conditioned media by ultracentrifugation and used to stimulate normal rat kidney interstitial fibroblasts (NRK-49F), followed by incubating with cisplatin (25 μg/ml) for 24 h. **b** Western blotting analyses demonstrate protein expression of CD63, TSG101 and Calnexin in TGF-β1-treated HK-2 cells. Numbers (1–3) indicate each individual culture plate in a given group. **c** Representative transmission electron microscopy (TEM) shows the exosomes isolated from conditioned media of HK-2 cells. Scale bar, 200 nm. **d** Western blotting analyses show induction of CD63 and TSG101 expression in EVs isolated from TGF-β1-treated HK-2 cells. Numbers (1–3) indicate EVs isolated from each individual treatment of HK-2 cells in a given group. **e** Average size distribution curve of exosomes released from HK-2 cells with or without TGF-β1 treatment. Exosomes size was determined by Nanoparticle Tracking Assay (NTA). Three 60 s videos were recorded for each sample, and NTA analysis settings kept constant between samples. The average concentration of vesicles was plotted against their size, with the black lines and the color areas representing the fitting curve and the error bar, respectively. **f** Fluorescent staining confirms the intracellular uptake of HK-2 cell-derived exosomes by NRK-49F cells. HK-2 cells were incubated and labeled with Dil-C18 (red), then exosomes were isolated. HK-2 cell-derived exosomes (30 μg/ml) were incubated with NRK-49F cells for 24 h, followed by immunofluorescence staining for α-tubulin (green). Arrows indicate HK-2 cell-derived exosomes. Scale bar, 25 µm. **g** Quantitative data of Dil^+^ fluorescence intensity in NRK-49F cells. (*n* = 3). **h**, **i** Representative FACS analyses (**h**) and quantitative data (**i**) show that exosomes derived from TGF-β1-treated HK-2 cells (CTL-Exo or TGFβ-Exo, 30 μg/ml) reduced cisplatin-caused fibroblast apoptosis. The PE-labeled Annexin V-positive cells were counted by flow cytometry. ^**^P < 0.01, ^††^*P* < 0.01 (*n* = 3). **j** Representative micrographs show TUNEL-positive cells in different groups as indicated. Scale bar, 50 µm. **k** Graphic presentation shows the quantitative determination of TUNEL-positive fibroblast cells in different groups. Each point indicates one of three different random fields of view in one micrograph. ^**^P < 0.01, ^††^*P* < 0.01 (*n* = 3). **l** Western blotting analyses demonstrate protein expression of p53, cleaved caspase-3, FasL, FADD, PARP-1, fibronectin and α-SMA after various treatments in NRK-49F cells. Numbers (1–3) indicate each individual treatment in a given group. **m** Representative micrographs show immunofluorescence staining of fibronectin in different groups as indicated. Arrows indicate positive staining. Data presented as mean ± S.E.M. of three independent experiments. *p* values were calculated using unpaired Student’s *t* test for two groups comparison or Student-Newman-Kuels test for mutiple groups comparison.

We first examine the potential role of tubule-derived exosomes in regulating fibroblast apoptosis. To mimic the condition of injured/stressed tubular cells in fibrotic kidney, we treated human proximal tubular epithelial cells (HK-2) with TGF-β1, a master profibrotic factor [22]. After a short incubation with TGF-β1 for 6 h, HK-2 cells were washed and incubated in serum-free medium for another 48 h. Exosomes were then isolated from conditioned media and used to stimulate normal rat kidney interstitial fibroblasts (NRK-49F) (Fig. 1a). As shown in Fig. 1b and Supplementary Fig. S1a–c, TGF-β1 induced protein expression of CD63 and tumor susceptibility gene 101 (TSG101) in HK-2 cells, both of which are markers of exosomes [23–25], but not calnexin, which is a non-EVs marker. We next characterized the purified EVs from HK-2 cells by transmission electron microscopy (TEM), Western blotting and nanoparticle tracking analysis (NTA). As shown in Fig. 1c, the majority of isolated EVs were exosomes, as defined by their sizes, typical cup-shape and bilayer membrane. Meanwhile, the protein expression of CD63, TSG101 and calnexin indicated that TGF-β1 treatment stimulated the exosomes production in HK-2 cells (Fig. 1d and Supplementary Fig. S1d, e). Figure 1e shows the representative distribution plots of the exosomes released from HK-2 cells treated without (CTL-Exo) or with TGF-β1 (TGFβ-Exo), which also proved that the abundance and sizes of exosomes were increased in the EVs produced by TGF-β1-treated HK-2 cells. We next labeled the tubular cell-derived exosomes with Dil-C18, a fluorescent lipophilic cationic indocarbocyanine dye for a long-term tracing. As shown in Fig. 1f, g, when Dil-labeled exosomes (red) were incubated with NRK-49F cells, they were uptaken by fibroblasts, as outlined by staining for α-tubulin cytoskeleton (green). There was no significant difference in the uptake efficiency of fibroblasts to CTL-Exo and TGFβ-Exo, as measured by red fluorescence intensity. Furthermore, exosomes isolated from TGF-β1-treated HK-2 cells (TGFβ-Exo) were able to reduce cisplatin-triggered apoptosis of NRK-49F cells, as manifested by a decreased percentage of apoptotic cells measured by both annexin V-labeling flow cytometry (Fig. 1h, i) and terminal deoxynucleotidyl transferase–mediated dUTP nick end-labeling (TUNEL) staining (Fig. 1j, k). Similarly, TGFβ-Exo also reduced the expression of p53, cleaved caspase 3, Fas-associated death domain protein (FADD) and Poly (ADP-ribose) polymerase-1 (PARP-1) (Fig. 1l, and Supplementary Fig. S1f–i). Interestingly, TGFβ-Exo promoted NRK-49F cell activation and matrix production after cisplatin, as assessed by Western blotting for fibronectin and α-smooth muscle actin (α-SMA) expression (Fig. 1l, and Supplementary Fig. S1j, k) and immunostaining for fibronectin (Fig. 1m and Supplementary Fig. S1l). Together, these results indicate that injured tubular cell-derived exosomes are capable of protecting fibroblasts from apoptosis and promoting their activation in vitro.

### Blockade of exosome secretion from tubular cells induces fibroblast apoptosis in vitro

**Fig. 2:**
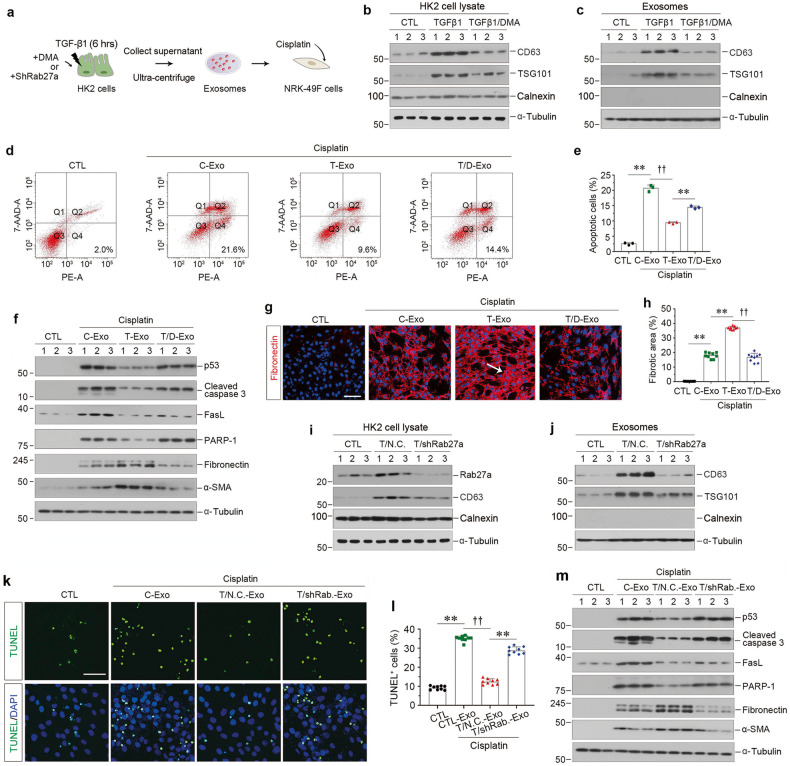
Inhibition of the exosome release from tubular cells promotes fibroblast apoptosis in vitro. **a** Experimental design. HK-2 cells were treated with TGF-β1 (2 ng/ml) for 6 h in the absence or presence of dimethyl amiloride (DMA) or shRab27a transfection, and then washed to remove all the treatments and continued to incubate for additional 48 h in serum-free medium. Exosomes were isolated from conditioned media by ultracentrifugation. **b** Representative Western blotting show CD63, TSG101 and Calnexin expression in HK-2 cells after DMA treatment. Numbers (1–3) indicate each individual treatment in a given group. **c** Representative Western blotting indicates that DMA attenuated the induction of CD63 and TSG101 expression in exosomes isolated from TGF-β1-treated HK-2 cells. Numbers (1–3) indicate exosomes isolated from each individual treatment in a given group. **d**, **e** Blockade of exosome generation by DMA abolished the cytoprotection elicited by exosomes derived from TGF-β1-treated HK-2 cells. Representative FACS analyses (**d**) and quantitative data (**e**) show the abundance of apoptotic cells in different groups. ^**^P < 0.01, ^††^*P* < 0.01 (*n* = 3). **f** Western blotting analyses demonstrate protein expression of p53, cleaved caspase-3, FasL, PARP-1, fibronectin and α-SMA after various treatments in NRK-49F cells. Numbers (1–3) indicate each individual treatment in a given group. **g** Representative micrographs show immunofluorescence staining of fibronectin in different groups as indicated. Arrows indicate positive staining. Scale bar, 50 µm. **h** Graphic presentation shows the quantitative determination of fibronectin-positive staining. Each point indicates one of three different random fields of view in one micrograph. ^**^P < 0.01, ^††^*P* < 0.01 (*n* = 3). **i** Western blotting indicates that knockdown of Rab27a reduced CD63 and Calnexin induction in TGF-β1-treated HK-2 cells. Numbers (1–3) indicate each individual treatment in a given group. **j** Western blotting shows the reduction of CD63 and TSG101 expression in exosomes isolated from TGF-β1-treated HK-2 cells after Rab27a knockdown. Numbers (1–3) indicate exosomes isolated from each individual treatment in a given group. **k** Representative micrographs show TUNEL-positive cells in different groups as indicated. Scale bar, 50 µm. **l** Graphic presentation shows the quantitative determination of TUNEL-positive fibroblast cells in different groups. Each point indicates one of three different random fields of view in one micrograph. ^**^P < 0.01, ^††^*P* < 0.01 (*n* = 3). **m** Western blotting analyses demonstrate protein expression of p53, cleaved caspase-3, FasL, PARP-1, fibronectin and α-SMA after various treatments in NRK-49F cells. Numbers (1–3) indicate each individual treatment in a given group. Data presented as mean ± S.E.M. of three independent experiments. *p* values were calculated using Student-Newman-Kuels test for multiple groups comparison.

To confirm the role for tubule-derived exosomes in fibroblast survival, we treated HK-2 cells with dimethyl amiloride (DMA), an inhibitor that blocks the biogenesis and release of exosomes [26–28], and then isolated exosomes from these cells to stimulate cisplatin-treated NRK-49F cells (Fig. 2a). As shown in Fig. 2b, c and Supplementary Fig. S2a–e, DMA inhibited TGF-β1-induced CD63 and TSG101 expression in HK-2 cells and exosomes purified from HK-2 cells, suggesting the production of exosomes were blocked by DMA. We then found that blockade of exosomes secretion restored the cisplatin-mediated fibroblast apoptosis, as measured by flow cytometry (Fig. 2d, e). Blockade of exosome production by DMA also upregulated the expression of p53, cleaved caspase 3, Fas ligand (FasL) and PARP-1, which were hampered after incubation with TGFβ-Exo, in NRK-49F cells (Fig. 2f and Supplementary Fig. S2f–i). On the contrary, inhibition of exosomes by DMA prevented NRK-49F cell activation, as demonstrated by fibronectin and α-SMA protein expression (Fig. 2f and Supplementary Fig. S2j, k), as well as immunostaining of fibronectin (Fig. 2g, h).

We also evaluated the role of tubule-derived exosomes in fibroblast survival by knocking down Rab27a, a small GTPases implicated in regulating exosome production and secretion [26, 29, 30], in HK-2 cells by transfecting with short hairpin RNA (shRNA) vector encoding the interference sequence of Rab27a (pLVX-shRab27a) or scrambled shRNA (Negative control, N.C.) (Fig. 2a). Knockdown of Rab27a decreased CD63 expression in HK-2cells after incubation with TGF-β1 (Fig. 2i and Supplementary Fig. S2l, m), suggesting that Rab27a mediates exosome production. The reduction of CD63 and TSG101 expression in exosomes extracted from the same number of HK-2 cells confirmed that Rab27a depletion suppressed exosome production (Fig. 2j and Supplementary Fig. S2o, p). Similar to DMA treatment, blockade of exosome production by Rab27a depletion induced NRK-49F cell apoptosis, as detected by TUNEL staining (Fig. 2k, l) and promoted the expression of multiple apoptosis-regulatory proteins, such as p53, cleaved caspase 3, FasL and PARP-1 (Fig. 2m and Supplementary Fig. S2q–t). However, TGFβ-Exo-stimulated fibroblast activation was inhibited by silencing Rab27a, as assessed by fibronectin and α-SMA expression (Fig. 2m and Supplementary Fig. S2u, v). Therefore, these data illustrate that exosomes released by TGF-β1-treated tubular cells play a critical role in mediating fibroblast survival and activation.

### Tubular cell-derived exosomes promote kidney fibrosis by augmenting fibroblast survival in vivo

**Fig. 3:**
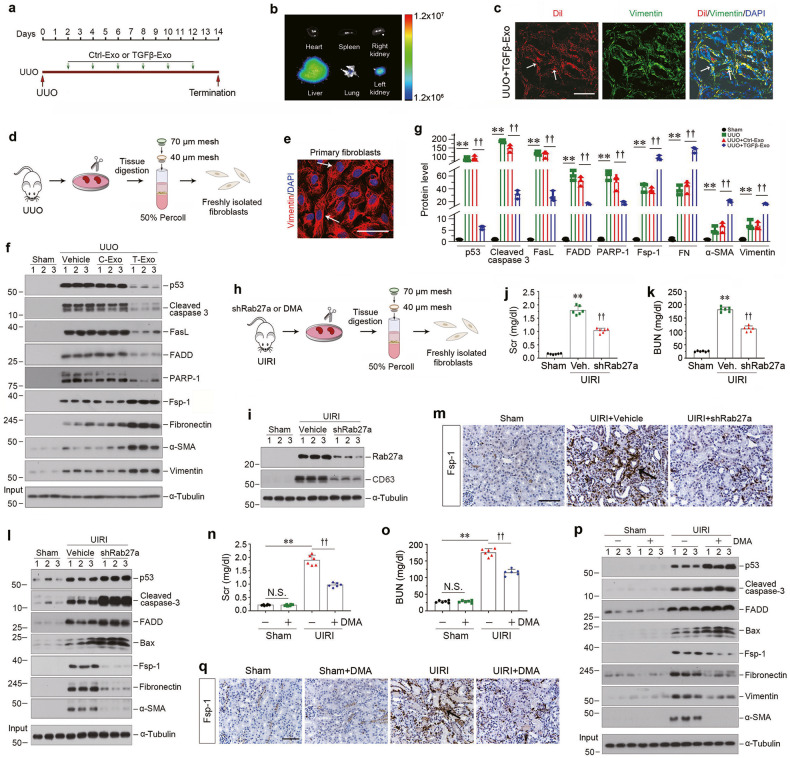
Tubular cells-derived exosomes attenuate renal fibroblast apoptosis in vivo. **a** Experimental design. Green arrows indicate the time points when HK-2 cells-derived exosomes (CTL-Exo or TGFβ-Exo, 200 μg) were injected intravenously. **b** Imaging of fluorescence intensity show Dil-C18-labeled exosomes in indicated organs at 1 day after the last intravenous injection. Dil-C18-labeled exosomes (red) isolated from TGF-β1-treated HK-2 cells were injected through the tail vein into UUO mice. **c** Representative images show Dil-C18-labeled exosomes in mouse kidney at 1 day after the last intravenous injection. Kidney cryosections were visualized for tracking Dil-C18-labeled exosomes (red) and activated fibroblasts (green) by immunofluorescence staining of vimentin. Arrows indicate positive labeling and staining. Scale bar, 50 µm. **d** Diagram shows the procedure of renal primary fibroblasts isolation. **e** Representative micrograph shows the vimentin expression of primary fibroblasts isolated from kidney after UUO. Arrows indicate positive staining. Scale bar, 50 µm. **f**, **g** Western blot analyses show the protein level changes of p53, cleaved caspase-3, FasL, FADD, PARP-1, Fsp-1, fibronectin, α-SMA and vimentin in different groups of primary fibroblasts isolated from kidney at 14 days after UUO. Representative Western blot (**f**) and quantitative data (**g**) are presented. Numbers (1–3) indicate a pool of primary fibroblasts isolated from two animals. ^**^P < 0.01, ^††^*P* < 0.01 (*n* = 3). **h** Diagram shows the experimental design in UIRI model. **i** Western blot analyses show renal expression of Rab27a and CD63 in different groups as indicated. Numbers (1–3) indicate each individual animal in a given group. **j** Graphic presentation shows the serum creatinine levels in different groups at 11 days after IRI. ^**^P < 0.01, ^††^*P* < 0.01 (*n* = 6). **k** Graphic presentation shows the blood urea nitrogen levels in different groups at 11 days after IRI. ^**^P < 0.01, ^††^*P* < 0.01 (*n* = 6). **l** Western blot analyses show the protein levels of p53, cleaved caspase-3, FADD, Bax, Fsp-1, fibronectin and α-SMA in different groups of primary fibroblasts isolated from kidney at 11 days after UIRI. Numbers (1–3) indicate a pool of primary fibroblasts isolated from two animals. **m** Representative micrographs show Fsp-1-positive cells in different groups as indicated. Scale bar, 50 µm. **n** Serum creatinine levels in different groups of mice as indicated. ^**^P < 0.01, ^††^*P* < 0.01, N.S. not significant (*n* = 6). **o** Blood urea nitrogen levels in different groups of mice as indicated. ^**^P < 0.01, ^††^*P* < 0.01, N.S. not significant (*n* = 6). **p** Western blot analyses show the protein levels of p53^,^ cleaved caspase-3, FADD, Bax, Fsp-1, fibronectin, vimentin and α-SMA in primary fibroblasts isolated from UIRI after DMA treatment. Numbers (1–3) indicate a pool of primary fibroblasts isolated from two animals. **q** Representative micrographs show Fsp-1-positive cells in different groups as indicated. Scale bar, 50 µm. Data presented as mean ± S.E.M. of three independent experiments. *p* values were calculated using Student-Newman-Kuels test for multiple groups comparison.

To investigate the role of tubule-derived exosomes in kidney fibrosis in vivo, we carried out animal studies using UUO model by injecting TGFβ-Exo isolated from TGF-β1-treated HK-2 cells, as shown in Fig. 3a. UUO mice were given same amounts of CTL-Exo or TGFβ-Exo at 2, 4, 6, 8, 10 and 12 days. We first examined whether exogenous exosomes can be delivered into kidneys by tail vein injection. As shown in Fig. 3b, Dil-C18-labeled exosomes could only be observed in the liver and obstructed kidney in vivo, whereas there was no noticeable change in other organs and healthy contralateral kidney, suggesting that exosomes can preferentially target the injured kidney. Co-staining revealed that Dil-C18-labeled exosomes were associated with fibroblast marker vimentin (Fig. 3c). We then examined the effects of these treatments on renal damage and fibrotic lesions. As shown in Supplementary Fig. S3a, TGFβ-Exo aggravated damage of the obstructed kidneys at 14 days after UUO, compared to CTL-Exo, whereas CTL-Exo caused no significant effect in the obstructed kidneys, as detected by Kim-1 mRNA level. Masson’s trichrome staining (MTS) and immunostaining revealed that TGFβ-Exo further exacerbated collagen deposition, and increased renal fibronectin, platelet-derived growth factor receptor-β (PDGFR-β) and α-SMA expression in the obstructed kidneys, as well as fibroblast specific protein 1 (Fsp-1) (Supplementary Fig. S3b, c–f).

To quantitatively assess the effect of TGFβ-Exo on renal interstitial fibroblasts in vivo, we isolated primary kidney fibroblasts via differential sieving coupled with Percoll centrifugation approach (Fig. 3d). The primary cells isolated were stained positively for vimentin, confirming the identity of kidney interstitial fibroblasts, and the purity of primary fibroblasts by this approach was quite high (Fig. 3e). We found that UUO induced the expression of apoptosis-related proteins, such as p53, cleaved caspase 3, FasL, FADD and PARP-1 in primary renal fibroblasts (Fig. 3f, g). However, injections of exogenous TGFβ-Exo largely abolished these inductions, compared to CTL-Exo (Fig. 3f, g). Moreover, TGFβ-Exo further aggravated UUO-induced fibroblast activation, as manifested by Fsp-1, fibronectin, α-SMA and vimentin expression (Fig. 3f, g). These results suggest that exogenous TGFβ-Exo prevents renal fibroblasts from apoptosis and promotes their activation in vivo.

### Blockade of exosome secretion aggravates fibroblast apoptosis in mice

To further confirm the role of exosomes in fibroblast survival and apoptosis, we carried out experiments to block the release of exosomes by knocking down Rab27a in mouse model of UIRI. The experimental design for UIRI model is presented in Supplementary Fig. 4a and Fig. 3h. At 4 days after UIRI, mice were injected with pLVX-shRab27a or scrambled shRNA through a hydrodynamic-based gene delivery approach [20, 31]. Silencing Rab27a reduced CD63 in the kidney after IRI (Fig. 3i and Supplementary Fig. S4b, c). We found that knockdown of Rab27a alleviated kidney injury with a reduced serum creatinine and blood urea nitrogen (Fig. 3j, k). Western blotting showed that depletion of Rab27a further augmented the expression of p53, cleaved caspase 3, FADD and Bax in primary renal fibroblasts (Fig. 3l and Supplementary Fig. S4e–h). However, knockdown of Rab27a hampered primary renal fibroblasts activation, as manifested by a reduced Fsp-1, fibronectin and α-SMA expression (Fig. 3l and Supplementary Fig. S4i–k). Similar results also were observed when kidney sections were immunostained for Fsp-1 protein (Fig. 3m), collagen deposition and Kim-1 protein (Supplementary Fig. S4l–o).

We also carried out another set of experiments to inhibit exosome production and secretion by DMA in UIRI model. As shown in Supplementary Fig. S5a–c, DMA inhibited CD63 expression. We found that DMA reduced serum creatinine and blood urea nitrogen levels at 11 days after UIRI (Fig. 3n, o), as well as Kim-1 mRNA expression (Supplementary Fig. S5l). Blockade of exosomes biogenesis and secretion by DMA also increased the expression of p53, cleaved caspase 3, FADD and Bax in primary renal fibroblasts isolated from UIRI kidneys (Fig. 3p and Supplementary Fig. S5d–g), whereas DMA abolished Fsp-1, fibronectin, vimentin and α-SMA induction (Fig. 3p and Supplementary Fig. S5h–k). Furthermore, blockade of exosome secretion by DMA ameliorated renal interstitial fibrotic lesions, as illustrated by Masson’s trichrome staining, Picrosirius red staining, and immunostaining for fibronectin (Supplementary Fig. S5n–p), and decreased renal fibroblast population, as observed by Fsp-1 immunostaining (Fig. 3q and Supplementary Fig. S5m). Of note, DMA did not cause any kidney abnormality in normal mice (Supplementary Fig. S5n–p). Taken together, blockade of exosome secretion either by knockdown of Rab27a or DMA promotes renal fibroblasts apoptosis, reduces fibroblast population and mitigate renal fibrosis in an established kidney injury.

### TNFAIP8 is enriched in the kidney exosomes in CKD

**Fig. 4:**
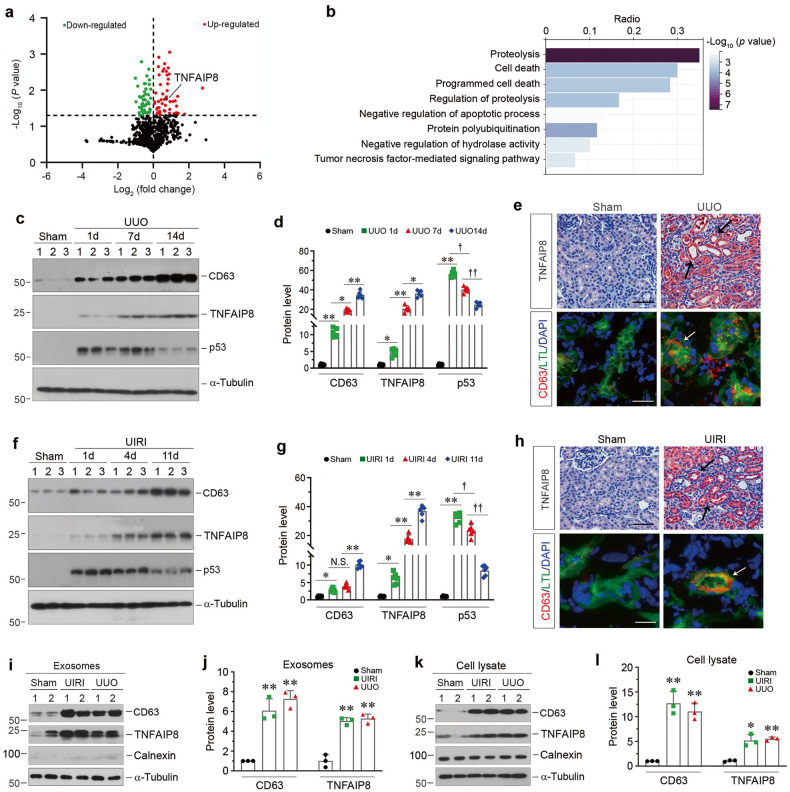
TNFAIP8 is enriched in tubular cells-derived exosomes and upregulated in various models of CKD. **a** Volcano plot shows the differentially expressed proteins of exosomes released from HK-2 cells without or with TGF-β1 treatment. **b** Gene ontology (GO) enrichment analysis reveals that several biological processes were enriched as indicated. **c**, **d** Western blot analyses show the renal expression of CD63, TNFAIP8 and p53 at different time points after UUO. Representative Western blot (**c**) and quantitative data (**d**) are presented. Numbers (1–3) indicate each individual animal in a given group. ^*^P < 0.05, ^**^P < 0.01, ^†^*P* < 0.05, ^††^*P* < 0.01 (*n* = 6). **e** Representative micrographs of immunohistochemical and immunofluorescence staining show tubular TNFAIP8 and CD63 expression in the kidney after UUO. Double immunofluorescence staining demonstrates the generation of exosomes predominantly in the proximal tubular epithelium. Kidney sections were co-stained for CD63 (Red) and lotus tetragonolobus lectin (LTL) (Green). Arrow indicates positive staining. Scale bar, 50 µm. **f**, **g** Western blot analyses show the renal expression of CD63, TNFAIP8 and p53 at different time points after UIRI. Representative Western blot (**f**) and quantitative data (**g**) are presented. Numbers (1–3) indicate each individual animal in a given group. ^*^P < 0.05, ^**^P < 0.01, ^†^*P* < 0.05, ^††^*P* < 0.01^,^ N.S. not significant (*n* = 6). **h** Representative micrographs of immunohistochemical and immunofluorescence staining show tubular TNFAIP8 and CD63 expression in the kidney after UIRI. Kidney sections were co-stained for CD63 (Red) and lotus tetragonolobus lectin (LTL) (Green). Arrow indicates positive staining. Scale bar, 50 µm. **i**, **j** Western blot analyses show the presence of CD63 and TNFAIP8 proteins in the exosomes isolated from kidneys after UIRI or UUO. Representative Western blot (**i**) and quantitative data (**j**) are presented. Numbers (1 and 2) indicate a pool of exosomes isolated from two animals. ^**^P < 0.01 versus sham controls (*n* = 3). **k**, **l** Western blot analyses show the presence of CD63, TNFAIP8, Calnexin proteins in the primary cells isolated from kidneys after UIRI or UUO. Representative Western blot (**k**) and quantitative data (**l**) are presented. Numbers (1 and 2) indicate a pool of primary cells isolated from two animals. ^*^P < 0.05, ^**^P < 0.01 versus sham controls (*n* = 3). Data presented as mean ± S.E.M. of three or six independent experiments. *p* values were calculated using Student-Newman-Kuels test for multiple groups comparison.

To search for the constituent of tubule-derived exosomes that is responsible for protecting fibroblast against apoptosis, exosomal proteins were profiled by proteomic analysis using liquid chromatography with tandem mass spectrometry (LC-MS/MS). By analyzing the differentially expressed proteins between the exosomes isolated from HK-2 cells treated with TGF-β1 (TGFβ-Exo) or without (CTL-Exo), we identified 101 differentially expressed proteins, of which 53 were downregulated and 48 were upregulated in TGFβ-Exo, compare with CTL-Exo (Fig. 4a and Supplementary Table S3). Furthermore, GO enrichment analysis showed that these proteins were enriched in proteolysis, programmed cell death and protein polyubiquitination pathways (Fig. 4b). In particular, tumor necrosis factor-α-induced protein 8 (TNFAIP8), which acts as an anti-apoptotic and oncogenic molecule [32, 33], was identified as one of the significantly upregulated proteins in TGFβ-Exo (Fig. 4a), suggesting that exosomal-TNFAIP8 may play a role in regulating fibroblast apoptosis.

We then investigated TNFAIP8 expression in mouse models of CKD induced by UUO and UIRI, respectively. Time-course study revealed that TNFAIP8 started to increase as early as day 1 in UUO models, and continued to increase along with the progression of renal fibrosis, which was concomitant with CD63 induction (Fig. 4c, d). Interestingly, renal expression of p53 was upregulated at day 1 after UUO, and then negatively correlated with TNFAIP8 in diseased kidneys after UUO (Fig. 4c, d). Immunostaining for TNFAIP8 revealed that this protein was predominantly localized in renal tubular epithelium (Fig. 4e). Co-staining for CD63 and *lotus tetragonolobus lectin* (LTL), a specific marker for proximal tubule, showed that proximal tubular epithelium was the major source of exosomes in diseased kidneys (Fig. 4e). Similar results were obtained when we examined the expression and localization of TNFAIP8, CD63 and p53 in UIRI model (Fig. 4f–h). Furthermore, we isolated exosomes from the diseased kidneys after UUO or UIRI, and Western blot analyses confirmed the upregulated CD63 and TNFAIP8 proteins in the isolated exosomes (Fig. 4i, j) and primary cells (Fig. 4k, l), suggesting that TNFAIP8 is a constituent capsulated in renal tubule-derived exosomes.

### TNFAIP8 capsulated in tubular exosomes protect fibroblasts from apoptosis

**Fig. 5:**
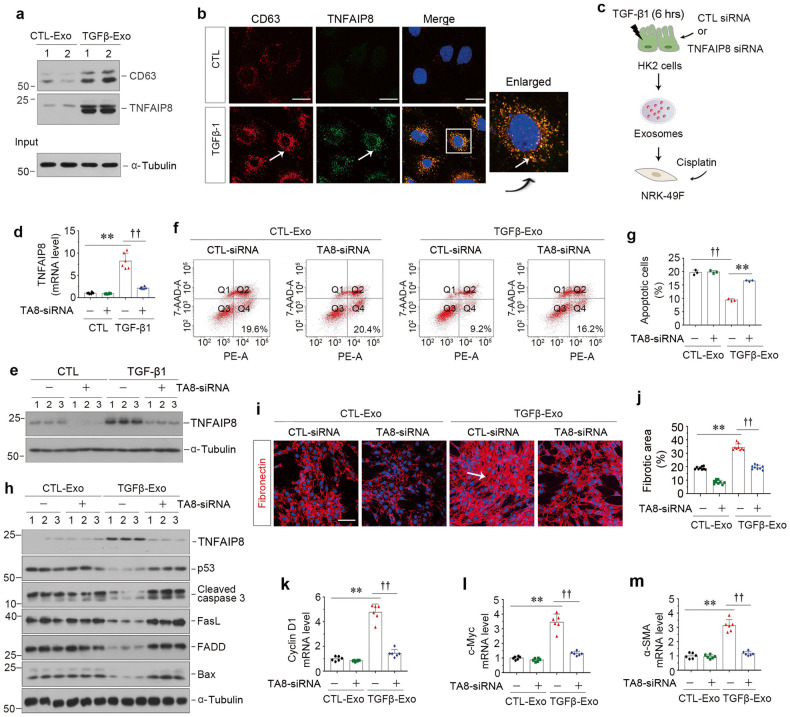
TNFAIP8 from tubule-derived exosomes plays a major role in protecting fibroblasts from apoptosis in vitro. **a** Western blot analyses show the presence of TNFAIP8 protein in the exosomes isolated from TGF-β1-treated HK-2 cells. Exosomes prepared from the equal amounts of HK-2 cells after TGF-β1 treatment were lysed and immunoblotted with antibodies against CD63 and TNFAIP8, respectively. Numbers (1 and 2) indicate each individual treatment in a given group. **b** Double immunofluorescence staining demonstrates colocalization of CD63 (Red) and TNFAIP8 (Green) in HK-2 cells after TGF-β1 treatment. Scale bar, 10 μm. **c** Experimental design showing knockdown of TNFAIP8 in HK-2 cells prior to exosomes collection of conditioned media. **d**, **e** Knockdown of TNFAIP8 abolished its expression after TGF-β1 treatment in HK-2 cells. qPCR (**d**) and Western blotting (**e**) show mRNA and protein levels of TNFAIP8 in different groups as indicated. ^**^P < 0.01, ^††^*P* < 0.01 (*n* = 6). Representative FACS analyses (**f**) and quantitative data (**g**) show apoptotic cells after various treatments in NRK-49F cells. ^**^P < 0.01, ^††^*P* < 0.01 (*n* = 3). **h** Western blot analyses demonstrate protein expression of TNFAIP8, p53, cleaved caspase-3, FasL, FADD, Bax in different groups of NRK-49F cells. Numbers (1–3) indicate each individual treatment in a given group. **i** Representative micrographs show immunofluorescence staining of fibronectin in different groups as indicated. Arrows indicate positive staining. Scale bar, 50 µm. **j** Graphic presentation shows the quantitative determination of fibronectin-positive area in different groups as indicated. Each point indicates one of three different random fields of view in one micrograph. ^**^P < 0.01, ^††^*P* < 0.01 (*n* = 3). **k**–**m** Graphic presentations show the relative level of cyclin D1 (**k**), c-myc (**l**) and α-SMA (**m**) mRNA measured by qPCR after various treatments in NRK-49F cells. ^**^P < 0.01, ^††^*P* < 0.01 (*n* = 6). Data presented as mean ± S.E.M. of three or six independent experiments. *p* values were calculated using Student-Nwman-Kuels test for multiple groups comparison.

We next assessed whether TNFAIP8 is involved in promoting exosomes-mediated fibroblast survival. As shown in Fig. 5a, TGF-β1 induced the biogenesis and release of exosomes from HK-2 cells, as assessed by CD63 expression, and these exosomes (TGFβ-Exo) contained abundant TNFAIP8. Immunofluorescence staining also revealed the induction of CD63 and TNFAIP8 by TGF-β1 in HK-2 tubular cells (Fig. 5b), in which co-localization of CD63 and TNFAIP8 was evident (Fig. 5b, enlarged image), demonstrating that TNFAIP8 can be capsulated by exosomes.

To ascertain the role of exosomes-derived TNFAIP8 in mediating fibroblast survival, we knocked down TNFAIP8 expression in HK-2 cells by transfecting with TNFAIP8-specific siRNA (TNFAIP8-siRNA) or scrambled siRNA (CTL-siRNA). As shown in Fig. 5c, exosomes were collected from HK-2 cells treated with or without TGF-β1 and used to stimulate cisplatin-treated NRK-49F fibroblasts. As shown in Fig. 5d, e and Supplementary Fig. S6a, knockdown of TNFAIP8 in HK-2 cells inhibited the induction of TNFAIP8 mRNA and protein expression by TGF-β1. Annexin V-labeling flow cytometry revealed that depletion of TNFAIP8 in HK-2 cells abolished the ability of TGFβ-Exo to prevent cisplatin-mediated apoptosis in NRK-49F recipient cells (Fig. 5f, g). Similar results were obtained when we analyzed multiple apoptosis-regulatory proteins by Western blotting. As shown in Fig. 5h and Supplementary Fig. S6b–g, deprivation of TNFAIP8 in HK-2 cells restored TGFβ-Exo-blocked expression of p53, cleaved caspase 3, FasL, FADD and Bax in NRK-49F cells. Conversely, TNFAIP8 depletion in HK-2 cells suppressed TGFβ-Exo-mediated stimulation of NRK-49F cell proliferation and activation, as demonstrated by fibronectin, cyclin D1, c-Myc and α-SMA expression (Fig. 5i–m). These results suggest that TNFAIP8 capsulated in tubular cell-derived exosomes plays a crucial role in mediating fibroblast survival and activation.

### TNFAIP8 abundance controls the size of fibroblast population and kidney fibrosis in vivo

**Fig. 6:**
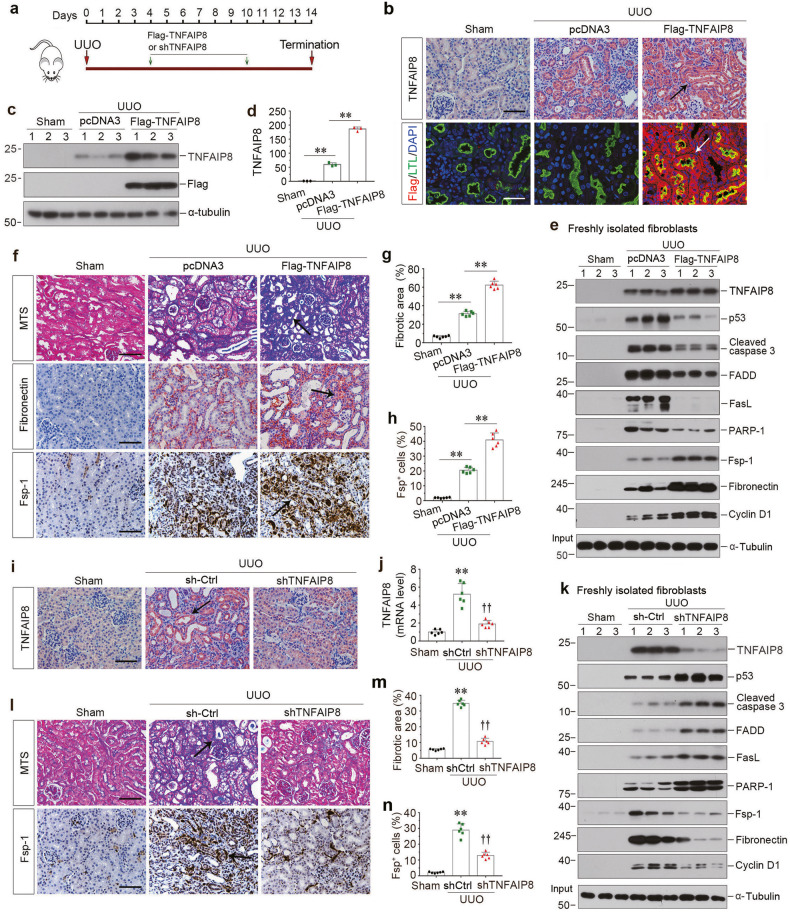
Over-expression or knockdown of TNFAIP8 inhibits or promotes renal interstitial fibroblast apoptosis in vivo. **a** Diagram shows the experimental design. Green arrow indicates the injection of Flag-TNFAIP8 expression vector or shTNFAIP8 knockdown plasmid. **b** Representative micrographs of immunohistochemical and immunofluorescence staining show an increased tubular TNFAIP8 expression after injection of Flag in the kidney after UUO. Arrows indicate positive staining. Scale bar, 50 µm. **c** Representative Western blotting show induction of TNFAIP8 expression in UUO kidneys by using different antibodies against TNFAIP8 and Flag, respectively. **d** Quantitative data of TNFAIP8 levels in different groups as indicated. ^**^P < 0.01 (*n* = 3). **e** Western blot analyses show the protein levels of TNFAIP8, p53, cleaved caspase-3, FasL, FADD, PARP-1, Fsp-1, fibronectin and cyclinD1 in different groups of primary fibroblasts isolated from kidney at 14 days after UUO. Numbers (1–3) indicate a pool of primary fibroblasts isolated from two animals. **f** Representative micrographs show collagen deposition, fibronectin and Fsp-1 expression in different groups as indicated. Paraffin sections were subjected to Masson’s trichrome staining (upper), immunostaining for fibronectin (middle) and Fsp-1 (bottom), respectively. Arrows indicate positive staining. Scale bar, 50 µm. Graphic presentations show the quantitative determination of fibronectin (**g**) and Fsp-1 (**h**) positive staining. ^**^P < 0.01 (*n* = 6). **i** Representative micrographs of immunohistochemical staining show tubular TNFAIP8 expression after injection of shTNFAIP8 plasmid after UUO. Arrows indicate positive staining. Scale bar, 50 µm. **j** Graphic presentations show the relative level of TNFAIP8 mRNA measured by qPCR after various treatments in UUO kidneys. ^**^P < 0.01 versus sham controls, ^††^*P* < 0.01 versus UUO injected with shCtrl plasmid (*n* = 6). **k** Western blot analyses show the protein levels of TNFAIP8, p53, cleaved caspase-3, FasL, FADD, PARP-1, Fsp-1, fibronectin and cyclin D1 in different groups of primary fibroblasts isolated from kidney at 14 days after UUO. Numbers (1–3) indicate a pool of primary fibroblasts isolated from two animals. **l** Representative micrographs show collagen deposition and Fsp-1 expression in different groups as indicated. Paraffin sections were subjected to Masson’s trichrome staining (upper), immunostaining for Fsp-1 (bottom), respectively. Arrows indicate positive staining. Scale bar, 50 µm. Graphic presentations show the quantitative determination of collagen deposition (**m**) and Fsp-1 (**n**) positive staining. ^**^P < 0.01 versus sham controls, ^††^*P* < 0.01 versus UUO injected with shCtrl plasmid (*n* = 6). Data presented as mean ± S.E.M. of six independent experiments. *p* values were calculated using Student-Newman-Kuels test for multiple groups comparison.

To further define the function of TNFAIP8 in the pathogenesis of CKD, we manipulated TNFAIP8 expression in vivo by delivering overexpression plasmid (Flag-TNFAIP8) or shRNA (shTNFAIP8) knockdown plasmid, respectively. As shown in Fig. 6a, mice were injected intravenously with either Flag-TNFAIP8 or shTNFAIP8 plasmid vectors at 4 and 10 days after UUO. Immunostaining and Western blotting for TNFAIP8 and Flag in whole kidney lysates confirmed the efficacy of TNFAIP8 overexpression, as well as localization (Fig. 6b–d and Supplementary Fig. S7a, b). We then assessed the effects of TNFAIP8 overexpression on primary renal fibroblast population isolated from the obstructed kidneys. As shown in Fig. 6e and Supplementary Fig. S7c, k, overexpression of TNFAIP8 abolished the induction of several apoptosis-related proteins, such as p53, cleaved caspase 3, FADD, FasL and PARP-1, whereas activation and proliferation of primary renal fibroblasts was further enhanced, as measured by Fsp-1, fibronectin and cyclin D1 expression, in UUO mice. Immunostaining also revealed that TNFAIP8 overexpression aggravated UUO-induced kidney fibrosis, as manifested by an increased collagens and fibronectin deposition, as well as an enlarged fibroblast population (Fig. 6f–h).

We further confirmed the effect of TNFAIP8 on controlling fibroblast population by employing an opposite strategy via knocking down its expression. As shown in Fig. 6i, j, and Supplementary Fig. S7l–n, delivery of shTNFAIP8 reduced the expression of TNFAIP8 in both protein and mRNA levels in the obstructed kidney. Western blotting showed that TNFAIP8 depletion further elevated the increase of p53, cleaved caspase 3, FADD, FasL and PARP-1 in primary renal fibroblast population isolated from the obstructed kidneys (Fig. 6k and Supplementary Fig. S7o–s). However, the expression of Fsp-1, fibronectin and cyclin D1 was blocked after TNFAIP8 depletion (Fig. 6k and Supplementary Fig. S7t–w). Similar results were observed when kidney sections were immunostained for collagens deposition and Fsp-1 protein (Fig. 6l–n). Therefore, these data indicate that TNFAIP8 levels dictate the size and activation status of renal interstitial fibroblast population in vivo.

### Exosomal, but not endogenous, TNFAIP8 is essential for protecting fibroblasts from apoptosis

To further confirm that exosomal-TNFAIP8 plays an essential role in regulating fibroblast survival, we knocked down endogenous TNFAIP8 expression in recipient NRK-49F cells, and then treated cells with TGFβ-Exo or CTL-Exo (Supplementary Fig. S8a). Apoptotic cells were detected by TUNEL staining. As shown in Supplementary Fig. S8b, c, TGFβ-Exo inhibited apoptosis induced by cisplatin in NRK-49F cells, compared with CTL-Exo, whereas depletion of TNFAIP8 in recipient NRK-49F cells did not affect their apoptosis. Consistently, TNFAIP8 knockdown in NRK-49F cells did not restore the expression of various apoptosis-related proteins when treated with TGFβ-Exo, as demonstrated by p53, cleaved caspase 3, FADD and Bax expression (Supplementary Fig. S8d–h). Moreover, TNFAIP8 depletion in NRK-49F cells did not reduce TGFβ-Exo-induced expression of c-Myc, cyclin D1, fibronectin and α-SMA (Supplementary Fig. S8d, i–n). Collectively, these results suggest that tubule-derived exosomal, but not endogenous, TNFAIP8 plays a predominant role in controlling renal interstitial fibroblast survival and fate.

### Exosomal TNFAIP8 inhibits fibroblast apoptosis by promoting p53 ubiquitination

**Fig. 7:**
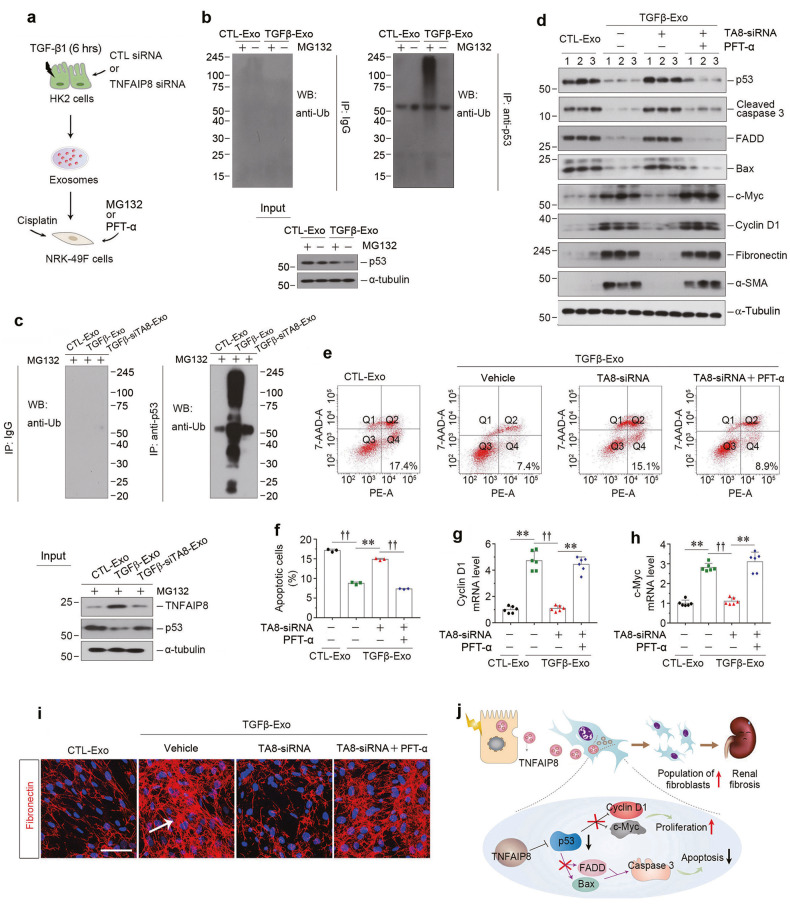
Exosomal-TNFAIP8 prevents fibroblast from apoptosis through blocking p53 signal pathway. **a** Diagram shows the experimental design. **b** NRK-49F cells incubated with exosomes (CTL-Exo or TGFβ-Exo, 30 μg/ml) were treated with or without MG132 for 6 h. Cell lysates were immunoprecipitated with anti-p53 antibody. The ubiquitination of the p53 was analyzed by western blotting using anti-ubiquitin antibody. **c** Co-immunoprecipitation assay show the status of p53 ubiquitination in NRK-49F cells after incubation with exosomes from different groups (30 μg/ml) as indicated and treated with MG132 for 6 h. **d** Western blotting analyses demonstrate protein expression of p53, cleaved caspase-3, FADD, Bax, c-myc, cyclin D1, fibronectin and α-SMA in different groups of NRK-49F cells. Numbers (1–3) indicate each individual treatment in a given group. Representative FACS analyses (**e**) and quantitative data (**f**) show the apoptotic cells after various treatments in NRK-49F cells. ^**^P < 0.01, ^††^*P* < 0.01 (*n* = 3). Graphic presentations show the relative level of cyclin D1 (**g**) and c-myc (**h**) mRNA measured by qPCR after various treatments in NRK-49F cells. ^**^P < 0.01, ^††^*P* < 0.01 (*n* = 6). **i** Representative micrographs show immunofluorescence staining of fibronectin in different groups as indicated. Arrows indicate positive staining. Scale bar, 50 µm. **j** Schematic presentation depicts the potential mechanism by exosomal-TNFAIP8 that prevents fibroblasts from apoptosis and promotes fibroblast activation through blocking p53 signal pathway. Data presented as mean ± S.E.M. of three or six independent experiments. *p* values were calculated using Student-Newman-Kuels test for multiple groups comparison.

We next delineated the mechanism of exosomal TNFAIP8 in protecting against fibroblast apoptosis. In view of that TNFAIP8 involves in the regulation of p53 ubiquitination in tumor cells [34], we speculated that exosomal-TNFAIP8 might also regulate p53 signaling to inhibit fibroblast apoptosis. To this end, we first treated NRK-49F cells with MG132 and incubated them without or with TNFAIP8-enriched exosomes (Fig. 7a). As shown in Fig. 7b, p53 ubiquitination was markedly increased in NRK-49F cells after incubating with TGFβ-Exo, compared with CTL-Exo. Notably, when TNFAIP8 was knocked down in TGF-β1-treated HK-2 cells, their exosomes devoid of TNFAIP8 could not promote p53 ubiquitination in NRK-49F cells (Fig. 7c). We then determined whether exosomal-TNFAIP8 led fibroblast survival resulted from the p53 activation by using PFT-α, a small molecule which has been widely used as a specific inhibitor of p53 transcription (Fig. 7a). As shown in Supplementary Fig. S9a–e, cisplatin-triggered apoptosis was attenuated by PFT-α in CTL-Exo treated NRK-49F cells, as measured by p53, cleaved caspase 3, FADD and FasL expression. Meanwhile, TGFβ-Exo also inhibited cisplatin-triggered NRK-49F cell apoptosis, which was abolished when TNFAIP8 was knocked down in HK-2 cells (Fig. 7d). However, inhibition of p53 by PFT-α, in NRK-49F cells clearly restored fibroblast survival, when incubating with TGFβ-Exo exosomes devoid of TNFAIP8 (Fig. 7d and Supplementary Fig. S9f–i). Similar results were observed when we detected apoptotic cells by flow cytometry analysis (Fig. 7e, f). On the contrary, PFT-α restored fibroblasts proliferation and activation caused by TGFβ-Exo, as shown by c-Myc, cyclin D1, fibronectin and α-SMA protein expression (Fig. 7d and Supplementary Fig. S9j–m), as well as cyclin D1 and c-Myc mRNA levels (Fig. 7g, h). Furthermore, immunostaining confirmed that PFT-α restored fibronectin deposition, which was inhibited by TNFAIP8-depleted TGFβ-Exo (Fig. 7i and Supplementary Fig. S9n). Taken together, as depicted in Fig. 7j, tubular cells play a critical role in controlling the survival, apoptosis and activation of interstitial fibroblasts through exosomes-mediated shuttling of TNFAIP8 to repress p53 signaling, thereby expanding fibroblast population in CKD.

## Discussion

After kidney injury, the injured or stressed tubular cells trigger fibroblasts activation and expansion by engaging epithelial-mesenchymal communication (EMC) via releasing exosomes and/or soluble factors [4, 8]. Emerging evidence indicates that exosomes, rather than soluble factors, play a predominant role in mediating EMC in renal fibrogenesis [11, 17, 18, 35, 36]. In the present study, we show herein that exosomes derived from the injured tubules control the size of interstitial fibroblast population in the fibrotic kidneys by inhibiting fibroblast apoptosis. This leads to an expansion of the matrix-producing fibroblast population and consequently aggravates kidney fibrosis. Consistent with this notion, blockade of exosome biogenesis and secretion by pharmacologic and genetic approaches decreases the pool size of fibroblasts by enhancing fibroblast apoptosis and repressing fibroblast proliferation in vivo and in vitro (Fig. 7j). These findings provide novel insights into the mechanism by which injured tubules dictate the fate of interstitial fibroblasts in the fibrotic kidney. Our studies also highlight a crucial role of exosomes, as a robust vehicle shuttling signals between cells, in mediating tubular epithelial-fibroblast communication during renal fibrogenesis.

Exosomes are nano-scale EVs which mediate intercellular communication through shuttling diverse substances to neighboring or remote target cells, thereby causing the phenotypic alterations of the recipient cells [13, 15]. Although almost every type of cell is capable of producing and secreting exosomes, the amounts and contents of exosomes from distinct cellular origins are very different, which are predominantly dictated by the physiological conditions, tissue microenvironment and biological context [11–13, 37]. An increase in exosome production is reported in a variety of CKD [11, 12, 38], and these exosomes can preferentially target different kidney cells including interstitial fibroblasts, tubular epithelial cells, macrophages and endothelial cells [17, 35, 36, 39, 40], thereby regulating the behaviors and phenotypes of the recipient cells and altering the trajectories of CKD progression.

One of the novel findings in the present study is that exosomes derived from injured tubular cells can promote fibroblast survival by inhibiting their apoptosis in response to death inducers. This conclusion is supported by several lines of evidence. First, tubular cell injury triggered by TGF-β1 leads to producing increased amounts of exosomes, which can be up-taken by interstitial fibroblasts (Fig. 1). Similarly, exosomes production is also upregulated in diseased kidneys, which occurs predominantly in tubular epithelium (Fig. 4). Second, the tubule-derived exosomes are capable of protecting fibroblasts against apoptosis by reducing the expression of multiple proapoptotic proteins such as p53, cleaved caspase-3, FasL, FADD and PARP-1 both in vitro and in vivo (Figs. 1 and 3). Third, disruption of the cellular machinery for exosome secretion by either pharmacologic or genetic means decreases cell survival of fibroblasts and kidney fibrosis (Fig. 2), suggesting an essential role for tubule-derived exosomes in controlling fibroblast fate during the pathogenesis of CKD.

Through proteomic profiling, the present study identifies TNFAIP8 as the key protein that is responsible for mediating tubular cells-controlled fibroblast survival. TNFAIP8 is enriched in the exosomes isolated from the injured kidneys (Fig. 4) and TGF-β1-treated HK-2 cells (Fig. 5), suggesting that it can be sorted into the cargo of exosomes after kidney injury. Consistently, TNFAIP8 is also markedly induced in renal tubular epithelium of diseased kidneys (Fig. 4). As a member of the TIPE family proteins, TNFAIP8 has been shown to have an anti-apoptotic and oncogenic potential and promote cell survival and drug resistance, cell proliferation, tumor growth and metastasis [32, 41–43]. Little is known, however, about its biological and pathological function in the kidneys, although it is induced in mesangial cells in diabetic nephropathy [44]. In this regard, it is a novel finding that exosomal-TNFAIP8 originated from renal tubules controls interstitial fibroblast fate, thereby determining the size of interstitial fibroblasts population. It should be stressed that knockdown of TNFAIP8 in the recipient NRK-49F fibroblasts did not affect their apoptosis or activation when stimulated with TGFβ-Exo (Supplementary Fig. S8), suggesting that exogenous and tubule-derived exosomal-, rather than endogenous fibroblast-intrinsic, TNFAIP8 plays a predominant role in mediating fibroblast survival.

The present study also delineates the potential mechanism underlying TNFAIP8 promotion of fibroblast survival by facilitating p53 degradation. Indeed, knockdown of TNFAIP8 in tubular epithelial cells reduces the TGFβ-Exo-mediated p53 ubiquitination in the recipient NRK-49F fibroblasts and abolishes their survival (Fig. 7). Furthermore, inhibition of p53 by small molecule inhibitor PFT-α restores the TGFβ-Exo-mediated protection of fibroblasts against apoptosis (Fig. 7), suggesting a critical role of p53 signaling in mediating TNFAIP8-mediated fibroblast protection. These findings are in harmony with earlier observations that TNFAIP8 regulates p53 signaling to reduce cell apoptosis in multiple types of cancer cells [34, 45, 46]. Consistently, incubation of fibroblasts with TGFβ-Exo reduces p53 protein and inhibits various proapoptotic proteins such as cleaved caspase-3, FADD and Bax (Fig. 7). It is worthwhile to point out that fibroblast apoptosis is inversely connected with fibroblast activation, as inhibition of apoptosis is often accompanied by activation and proliferation of fibroblasts, as manifested by an increased expression of c-Myc, cyclin D1, fibronectin and α-SMA (Fig. 7). This is not completely surprising, as a decreased p53 would lead to the de-repression of cyclin D1 and c-Myc, thereby causing fibroblast activation and proliferation (Fig. 7j).

Apart from TNFAIP8, several previous studies indicate that other mediators in the tubule-derived exosomes such as Shh protein [17], TGF-β1 mRNA [18] and miR-21 [47] may also be transferred to regulate kidney fibrosis by directly stimulating fibroblast activation. However, these mediators appear to be more relevant to fibroblast activation and proliferation, rather than fibroblast apoptosis. This notion is supported by the observations that over-expression of TNFAIP8 in vivo suppresses fibroblast apoptosis and aggravates kidney fibrosis in obstructive nephropathy, while knockdown of TNFAIP8 promotes fibroblast apoptosis and mitigates fibrotic lesions (Fig. 6). However, we cannot completely exclude the possibility that other mediators encapsulated in the tubule-derived exosomes besides TNFAIP8 may play a role in promoting fibroblast survival as well. Therefore, the complexity and relative importance of different components of the tubule-derived exosomes in fibrotic CKD deserve further investigation.

In summary, the present study indicates that exosome-mediated tubular-fibroblast communication plays a fundamental role in regulating fibroblast apoptosis and kidney fibrosis. We show that kidney injury triggers an increased expression of TNFAIP8 in tubular epithelium, which can be encapsulated by exosomes and delivered into interstitial fibroblasts. Furthermore, we demonstrate that the tubule-derived exosomal-TNFAIP8 promotes kidney fibrosis by targeting p53 ubiquitination and degradation, which leads to fibroblast survival and proliferation. These findings suggest a pivotal role of the tubular epithelial cells in dictating the fate of interstitial fibroblasts via exosomes-mediated cell-cell communication in diseased kidney. These studies pave a new avenue in developing therapeutics against CKD by targeting the biogenesis and secretion of exosomes.

### Supplementary information

supplementary file

Supplemental Material-wb

## Data Availability

The mass spectrometry proteomics data have been deposited to the ProteomeXchange Consortium (http://proteomecentral.proteomexchange.org) via the iProX partner repository with the dataset identifier PXD034545.
