# Supplementary material for: Kidney tubular epithelial cells control interstitial fibroblast fate by releasing TNFAIP8-encapsulated exosomes
Source: Cell Death Dis. 2023 Oct 12;14(10):672. doi: 10.1038/s41419-023-06209-w (PMC10570316; doi:10.1038/s41419-023-06209-w)
Supplement: Supplementary file 1 — supplementary file [file 41419_2023_6209_MOESM1_ESM.docx]

**Supplementary Figure legends**

**Figure S1. Tubule-derived exosomes plays a major role in mediating fibroblast apoptosis in vitro.** (a-c) Graphic presentations indicate the relative protein levels of CD63 (a), TSG101 (b) and Calnexin (c) in HK-2 cells without or with TGF-β1 treatment. **P* < 0.05, ***P* < 0.01 versus control (n = 6). (d, e) Graphic presentations demonstrate the relative protein levels of CD63 (a) and TSG101 (b) in exosomes isolated from HK-2 cells without or with TGF-β1 treatment. ***P* < 0.01 versus control (n = 6). (f-k) Graphic presentations show the relative protein levels of p53 (f), cleaved caspase-3 (g), FADD (h), PARP-1 (i), fibronectin (j) and α-SMA (k) after various treatments in NRK-49F cells. **P* < 0.05, ***P* < 0.01, †*P* < 0.05, ††*P* < 0.01 (n = 6). (l) Quantitative determination of fibronectin positive staining. Each point indicates the one of three different random field of view in one micrograph. ***P* < 0.01 (n = 3).

**Figure S2. Disruption of exosome release aggravates fibroblast apoptosis in vitro.** (a-c) Quantitative data show reduction of HK-2 cells CD63 (a), TSG101 (b) and Calnexin (c) expression after DMA treatment. ***P* < 0.01, ††*P* < 0.01 (n = 6). (d,e) Quantitative data show reduction of CD63 (d) and TSG101 (e) in exosomes isolated from DMA-treated HK-2 cells. ***P* < 0.01, ††*P* < 0.01 (n = 6). (f-k) Graphic presentations show the relative protein levels of p53 (f), cleaved caspase-3 (g), FasL (h), PARP-1 (i), fibronectin (j), and α-SMA (k) after various treatments in NRK-49F cells. **P* < 0.05, ***P* < 0.01, †*P* < 0.05, ††*P* < 0.01 (n = 6). (l-n) Quantitative data show shRab27a transfection disrupts expression of Rab27a (l), CD63 (m) and Calnexin (n) in HK-2 cells. ***P* < 0.01, ††*P* < 0.01 (n = 6). (o, p) Quantitative data show shRab27a transfection inhibits expression of CD63 (o) and TSG101 (p) in exosomes isolated from HK-2 cells. (q-v) Graphic presentations show the relative protein levels of p53 (q), cleaved caspase-3 (r), FasL (s), PARP-1 (t), fibronectin (u), and α-SMA (v) after various treatments in NRK-49F cells. ***P* < 0.01, ††*P* < 0.01 (n = 6).

**Figure S3. Tubular cells-derived exosomes aggravate fibroblast activation in vivo.** (a) Graphic presentation shows the relative level of Kim-1 mRNA in different groups as indicated. ***P* < 0.01, N.S., not significant (n = 6). (b) Representative micrographs show immunohistochemical staining of Masson’s trichrome staining for collagen deposition, fibronectin, PDGFR-β, α-SMA and fsp-1 in different groups as indicated. Arrows indicate positive staining. Scale bar, 50 µm. (c-f) Quantitative data on fibronectin (c), PDGFR-β (d), α-SMA (e) and fsp-1 (f) positive staining are presented. ***P* < 0.01, N.S., not significant (n = 6).

**Figure S4. Inhibition of exosome release by depleting Rab27a induces fibroblast apoptosis and protects against renal fibrosis in vivo.** (a) Diagram shows the experimental design. Red arrows indicate the time points of renal IRI surgery. Green arrow indicates the injection of shRab27a plasmid. (b, c) Quantitative data show reduction of IRI kidneys Rab27a (b) and CD63 (c) expression in different groups as indicated. ***P* < 0.01, ††*P* < 0.01 (n = 6). (d) Quantitative data on fsp-1 positive staining is presented. ***P* < 0.01, ††*P* < 0.01 (n = 6). (e-k) Graphic presentations show the relative protein levels of p53 (e), cleaved caspase-3 (f), FADD (g), Bax (h), fsp-1 (i), fibronectin (j) and α-SMA (k) in primary fibroblasts isolated from kidney after various treatments. ***P* < 0.01, ††*P* < 0.01 (n = 6). (l) Representative micrographs show immunohistochemical staining of Masson’s trichrome staining, Picrosirius red staining for collagen deposition, and Kim-1 in different groups as indicated. Arrows indicate positive staining. Scale bar, 50 µm. (m-o) Quantitative data on Masson’s trichrome staining (m), Picrosirius red staining (n), and Kim-1 (o) positive staining are presented. ***P* < 0.01, ††*P* < 0.01 (n = 6).

**Figure S5. Blockade of exosome secretion by dimethyl amiloride promotes fibroblast apoptosis and reduces renal fibrosis in vivo.** (a) Diagram shows the experimental design of UIRI. Red arrows indicate the time points undergoing UIRI, UNx and sacrifice, respectively. Green arrows indicate dimethyl amiloride (DMA) treatment (20 mg/kg body weight). (b, c) Western blotting (b) and quantitative data show reduction of IRI kidneys CD63 (c) expression after DMA treatment. Numbers (1 to 3) indicate each individual animal in a given group. ***P* < 0.01, ††*P* < 0.01, N.S., not significant (n = 6). (d-k) Graphic presentations show the relative protein levels of p53 (d), cleaved caspase-3 (e), FADD (f), Bax (g), fsp-1 (h), fibronectin (i), vimentin (j) and α-SMA (k) in primary fibroblasts isolated from kidney after various treatments. ***P* < 0.01, ††*P* < 0.01 (n = 6). (l) Graphic presentation shows the relative level of Kim-1 mRNA in different groups as indicated. ***P* < 0.01, ††*P* < 0.01, N.S., not significant (n = 6). (m) Quantitative data on fsp-1 positive staining are presented. ***P* < 0.01, ††*P* < 0.01, N.S., not significant (n = 6). (n) Representative micrographs show immunohistochemical staining of Masson’s trichrome staining, Picrosirius red staining for collagen deposition, and fibronectin in different groups as indicated. Arrows indicate positive staining. Scale bar, 50 µm. (o, p) Quantitative data on Masson’s trichrome staining (o) and Picrosirius red staining (p) positive area are presented. ***P* < 0.01, ††*P* < 0.01, N.S., not significant (n = 6).

**Figure S6. Exosomal-TNFAIP8 derived from tubular cells is sufficient and necessary for protecting fibroblast against apoptosis.** (a) Graphic presentation indicates the relative protein level of TNFAIP8 in HK-2 cells after TNFAIP8-siRNA transfection. ***P* < 0.01, ††*P* < 0.01, N.S. (n = 6). (b-g) Graphic presentations show the relative protein levels of TNFAIP8 (b), p53 (c), cleaved caspase-3 (d), FasL (e), FADD (f) and Bax (g) in different groups of NRK-49F cells as indicated. ***P* < 0.01, ††*P* < 0.01, N.S., not significant (n = 6).

**Figure S7. TNFAIP8 plays a major role in mediating fibroblast apoptosis in vivo.** (a) Graphic presentation indicates TNFAIP8 positive staining is presented after TNFAIP8 overexpression in UUO kidneys. ***P* < 0.01 (n = 6). (b) Quantitative data show induction of Flag in UUO kidneys. ***P* < 0.01, N.S., not significant (n = 3). (c-k) Graphic presentations show the relative protein levels of TNFAIP8 (c), p53 (d), cleaved caspase-3 (e), FADD (f), FasL (g), PARP-1 (h), fsp-1 (i), fibronectin (j) and cyclin D1 (k) in primary fibroblasts isolated from kidney after various treatments. **P* < 0.05, ***P* < 0.01, †*P* < 0.05, ††*P* < 0.01 (n = 3). (l) Graphic presentation indicates TNFAIP8 positive staining are presented after TNFAIP8 knockdown in UUO kidneys. ***P* < 0.01, ††*P* < 0.01 (n = 6). (m, n) Western blotting (m) and quantitative data (n) show expression of TNFAIP8 after TNFAIP8 knockdown in UUO kidneys. Numbers (1 to 3) indicate a pool of kidney tissue from two animals in a given group. ***P* < 0.01, †*P* < 0.05 (n = 3). (o-w) Graphic presentations show the relative protein levels of TNFAIP8 (o), p53 (p), cleaved caspase-3 (q), FADD (s), FasL (r), PARP-1 (s), fsp-1 (t), fibronectin (u) and cyclin D1 (w) in primary fibroblasts isolated from kidney after various treatments. ***P* < 0.01, ††*P* < 0.01 (n = 3).

**Figure S8. TNFAIP8 capsulated in tubular exosomes is sufficient for mediating fibroblast apoptosis rather than endogenous TNFAIP8.**  (a) Experimental design. (b, c) Representative FACS analyses (b) and quantitative data (c) show the abundance of apoptotic cells after various treatments in NRK-49F cells. ††*P* < 0.01, N.S., not significant (n = 3). (d-l) Representative Western blotting (d) analyses and quantitative data demonstrate protein expression of p53 (e), cleaved caspase-3 (f), FADD (g), Bax (h), c-myc (i), cyclin D1 (j), fibronectin (k) and α-SMA (l) in different groups of NRK-49F cells. Numbers (1 to 3) indicate each individual treatment in a given group. ***P* < 0.01, ††*P* < 0.01, N.S., not significant (n = 6). (m, n) Graphic presentations show the relative level of cyclin D1 (m) and c-myc (n) mRNA measured by qPCR after various treatments in NRK-49F cells. ***P* < 0.01, N.S., not significant (n = 6). (o) Representative micrographs show immunofluorescence staining of fibronectin in different groups as indicated. Arrows indicate positive staining. Scale bar, 50 µm. (p) Quantitative data of fibronectin positive staining are presented. Each point indicates the one of three different random field of view in one micrograph. ***P* < 0.01, N.S., not significant (n = 6).

**Figure S9. Exosomal-TNFAIP8 prevents fibroblast against apoptosis by promoting p53 ubiquitination.** (a-e) Representative Western blotting (a) analyses and quantitative data demonstrate protein expression of p53 (b), cleaved caspase-3 (c), FADD (d), FasL (e) in NRK-49F cells after incubation with exosomes from different groups (30 μg/ml) and treated with MG132 for 6 h. Numbers (1 to 3) indicate each individual treatment in a given group. ***P* < 0.01, ††*P* < 0.01, N.S., not significant (n = 6). (f-m) Graphic presentations show the relative protein levels of p53 (f), cleaved caspase-3 (g), FADD (h), Bax (i), c-Myc (j), cyclin D1 (k), fibronectin (l) and α-SMA (m) after various treatments in NRK-49F cells. ***P* < 0.01, ††*P* < 0.01 (n = 6). (n) Quantitative data of fibronectin positive staining are presented. Each point indicates one of three different random fields of view in one micrograph. ***P* < 0.01, ††*P* < 0.01 (n = 3).

**Supplementary Table S1. The sources of antibodies used in this study**

| **Antibodies** | **Catalogue number** | **Company** | **Location** |
| --- | --- | --- | --- |
| **Primary antibodies** | | | |
| anti-cleaved caspase-3 | #9664S | Cell Signaling Technology | Danvers, MA |
| anti-Fas-L | sc-6237 | Santa Cruz Biotechnology | Santa Cruz, CA |
| anti-Fibronectin | F3648 | Sigma-Aldrich | St. Louis, MO |
| anti-α-SMA | A2547 | Sigma-Aldrich | St. Louis, MO |
| anti-α-SMA | Ab5694 | Abcam | Cambridge, MA |
| anti-PAI-1 | AF3828 | R & D Systems | Minneapolis, MN |
| anti-FADD | #2782S | Cell Signaling Technology | Danvers, MA |
| anti-CD63 | Ab59479 | Abcam | Cambridge, MA |
| anti-CD63 | Ab217345 | Abcam | Cambridge, MA |
| anti-TSG101 | Ab83 | Abcam | Cambridge, MA |
| anti-Rab27a | Ab55667 | Abcam | Cambridge, MA |
| anti-Flag | F1804 | Sigma-Aldrich | St. Louis, MO |
| anti-Bax | sc-493 | Santa Cruz Biotechnolog | Santa Cruz, CA |
| anti-P53 | sc-126 | Santa Cruz Biotechnology | Santa Cruz, CA |
| anti-PARP-1 | #9542S | Cell Signaling Technology | Danvers, MA |
| anti-TNFAIP8 | Ab195810 | Abcam | Cambridge, MA |
| anti-TNFAIP8 | SAB1407255 | Sigma-Aldrich | St. Louis, MO |
| anti-Vimentin | ab6992 | Abcam | Cambridge, MA |
| anti-PDGFR-β | sc-432 | Santa Cruz Biotechnology | Santa Cruz, CA |
| anti-Fsp-1 | 07-2274 | Sigma-Aldrich | St. Louis, MO |
| anti-Cyclin D1 | #2922 | Cell Signaling Technology | Danvers, MA |
| anti-c-Myc | #5605S | Cell Signaling Technology | Danvers, MA |
| anti-Ubiquitin | #3933 | Cell Signaling Technology | Danvers, MA |
| anti-α-tubulin | RM2007 | Ray Antibody Biotech | Peachtree Corners, GA |
| anti-GAPDH | RM2002 | Ray Antibody Biotech | Peachtree Corners, GA |
| anti-Calnexin | #2433 | Cell Signaling Technology | Danvers, MA |
| **Secondary antibodies** | | | |
| Goat anti-mouse | BA1050 | Boster Biological Technology | Wuhan, China |
| Goat anti-rabbit | BA1054 | Boster Biological Technology | Wuhan, China |
| Rabbit anti-goat | BA1060 | Boster Biological Technology | Wuhan, China |
| Donkey Anti-Mouse | 715-065-150 | Jackson ImmunoResearch | West Grove, PA |
| Donkey Anti-Rabbit | 711-065-152 | Jackson ImmunoResearch | West Grove, PA |
| Donkey Anti-Mouse | 715-225-150 | Jackson ImmunoResearch | West Grove, PA |
| Donkey Anti-Rabbit | 711-165-152 | Jackson ImmunoResearch | West Grove, PA |

**Supplementary Table S2. Primers for qRT-PCR**

| **Gene name** | **Primer sequence** |
| --- | --- |
| *β-actin*  *c-Myc*  *Cyclin D1*  *Tnfaip8*  *α-SMA*  *Kim-1* | S:CAGCTGAGAGGGAAATCGTG A:CGTTGCCAATAGTGATGACC  S:TCCATCCTATGTTGCGGTCG  A:AACCGCTCCACATACAGTCC  S:TCAAGTGTGACCCGGACTG A:GACCAGCTTCTTCCTCCACTT  S:CCAAATCCATCGCCACCACCT A:TGGCCAGCTTGATGACTGTCT  S:CATCGTGTTGGATTCTGGGG  A:GTCACGAAGGAATAGCCACG  S:TATGTTGGCATCTGCATCGC  A:GAAGGCAACCACGCTTAGAG |

**Supplementary Table S3. Differentially expressed proteins**

| **Gene name** | **Fold change** | **Protein_size** | **Annotation** |
| --- | --- | --- | --- |
| AHSG | 0.162139 | 367 | alpha-2-HS-glycoprotein |
| CP | 0.309396 | 1065 | ceruloplasmin (ferroxidase) |
| SERPINA1 | 0.319557 | 418 | serpin peptidase inhibitor, clade A (alpha-1 antiproteinase, antitrypsin), member 1 |
| CLTA | 0.324192 | 248 | clathrin, light chain A |
| FGA | 0.332849 | 866 | fibrinogen alpha chain |
| IGLL5 | 0.333369 | 214 | immunoglobulin lambda-like polypeptide 5 |
| SERPINC1 | 0.337092 | 464 | serpin peptidase inhibitor, clade C (antithrombin), member 1 |
| C4B | 0.340441 | 1744 | complement component 4B (Chido blood group) |
| KNG1 | 0.372385 | 644 | kininogen 1 |
| HPX | 0.477329 | 462 | hemopexin |
| RPS25 | 0.520409 | 125 | ribosomal protein S25 |
| FGG | 0.523316 | 453 | fibrinogen gamma chain |
| APOA1 | 0.569241 | 267 | apolipoprotein A-I |
| TLN1 | 0.569599 | 2541 | talin 1 |
| PFKP | 0.587236 | 784 | phosphofructokinase, platelet |
| HIST1H4H | 0.588962 | 103 | histone cluster 1, H4h |
| RPS15A | 0.59082 | 130 | ribosomal protein S15a |
| HP1BP3 | 0.596701 | 553 | heterochromatin protein 1, binding protein 3 |
| ILF2 | 0.606439 | 390 | interleukin enhancer binding factor 2 |
| CSE1L | 0.611234 | 971 | CSE1 chromosome segregation 1-like |
| NQO1 | 0.614627 | 274 | NAD(P)H dehydrogenase, quinone 1 |
| CAND1 | 0.616087 | 1230 | cullin-associated and neddylation-dissociated 1 |
| RPS19 | 0.624494 | 145 | ribosomal protein S19 |
| GNAS | 0.625021 | 1037 | GNAS complex locus |
| PARP4 | 0.625035 | 1724 | poly (ADP-ribose) polymerase family, member 4 |
| PSMD13 | 0.626346 | 378 | proteasome (prosome, macropain) 26S subunit, non-ATPase, 13 |
| PSME2 | 0.631331 | 239 | proteasome (prosome, macropain) activator subunit 2 (PA28 beta) |
| PSMD3 | 0.631707 | 534 | proteasome (prosome, macropain) 26S subunit, non-ATPase, 3 |
| QSOX1 | 0.637477 | 747 | quiescin Q6 sulfhydryl oxidase 1 |
| MVP | 0.638751 | 893 | major vault protein |
| RPL31 | 0.641021 | 128 | ribosomal protein L31 |
| RPS7 | 0.641974 | 194 | ribosomal protein S7 |
| PSMD8 | 0.656323 | 350 | proteasome (prosome, macropain) 26S subunit, non-ATPase, 8 |
| PSMC3 | 0.657257 | 439 | proteasome (prosome, macropain) 26S subunit, ATPase, 3 |
| RPL26 | 0.661631 | 145 | ribosomal protein L26 |
| MATN2 | 0.661914 | 956 | matrilin 2 |
| NID2 | 0.665506 | 1375 | nidogen 2 (osteonidogen) |
| TUBA4A | 0.669399 | 448 | tubulin, alpha 4a |
| RPS3A | 0.673646 | 264 | ribosomal protein S3A |
| TEP1 | 0.678441 | 2627 | telomerase-associated protein 1 |
| CD9 | 0.680076 | 228 | CD9 molecule |
| CLIC1 | 0.680641 | 241 | chloride intracellular channel 1 |
| A2M | 0.683622 | 1474 | alpha-2-macroglobulin |
| PA2G4 | 0.68657 | 394 | proliferation-associated 2G4 |
| RPL3 | 0.689529 | 403 | ribosomal protein L3 |
| SSRP1 | 0.68961 | 709 | structure specific recognition protein 1 |
| STAT1 | 0.689834 | 750 | signal transducer and activator of transcription 1 |
| M6PR | 0.689971 | 277 | mannose-6-phosphate receptor (cation dependent) |
| RPS26 | 0.691783 | 115 | ribosomal protein S26 |
| CLIC4 | 0.692171 | 253 | chloride intracellular channel 4 |
| PPP2R1A | 0.692932 | 589 | protein phosphatase 2, regulatory subunit A, alpha |
| CDK1 | 0.695976 | 297 | cyclin-dependent kinase 1 |
| COPB2 | 0.697078 | 906 | coatomer protein complex, subunit beta 2 (beta prime) |
| AMBP | 0.698168 | 352 | alpha-1-microglobulin/bikunin precursor |
| RPL22 | 0.702634 | 128 | ribosomal protein L22 |
| HIST1H2BL | 0.702954 | 126 | histone cluster 1, H2bl |
| HIST1H1E | 0.705917 | 219 | histone cluster 1, H1e |
| IPO5 | 0.706139 | 1115 | importin 5 |
| RAN | 0.711954 | 216 | RAN, member RAS oncogene family |
| CUL4B | 0.714748 | 913 | cullin 4B |
| XRCC6 | 0.715167 | 609 | X-ray repair complementing defective repair in Chinese hamster cells 6 |
| RPS3 | 0.716574 | 243 | ribosomal protein S3 |
| EIF4A1 | 0.717449 | 406 | eukaryotic translation initiation factor 4A1 |
| OTUB1 | 0.718151 | 271 | OTU domain, ubiquitin aldehyde binding 1 |
| PSMC2 | 0.719659 | 433 | proteasome (prosome, macropain) 26S subunit, ATPase, 2 |
| DYNC1H1 | 0.719877 | 4646 | dynein, cytoplasmic 1, heavy chain 1 |
| SERPINH1 | 0.721035 | 418 | serpin peptidase inhibitor, clade H (heat shock protein 47), member 1, (collagen |
|  |  |  | binding protein 1) |
| SLC1A5 | 0.725294 | 541 | solute carrier family 1 (neutral amino acid transporter), member 5 |
| YWHAH | 0.725602 | 246 | tyrosine 3-monooxygenase/tryptophan 5-monooxygenase activation protein, eta polypeptide |
| RPS4X | 0.726597 | 263 | ribosomal protein S4, X-linked |
| CUL3 | 0.727808 | 768 | cullin 3 |
| SYNCRIP | 0.730753 | 623 | synaptotagmin binding, cytoplasmic RNA interacting protein |
| TNPO1 | 0.731172 | 898 | transportin 1 |
| LOXL2 | 0.733169 | 774 | lysyl oxidase-like 2 |
| GFPT1 | 0.734136 | 681 | glutamine--fructose-6-phosphate transaminase 1 |
| LDHB | 0.734232 | 334 | lactate dehydrogenase B |
| MCM2 | 0.735189 | 904 | minichromosome maintenance complex component 2 |
| PAICS | 0.73598 | 432 | phosphoribosylaminoimidazole carboxylase, phosphoribosylaminoimidazole succinocarboxamide synthetase |
| HSP90AB1 | 0.738881 | 724 | heat shock protein 90kDa alpha (cytosolic), class B member 1 |
| CCT5 | 0.739732 | 541 | chaperonin containing TCP1, subunit 5 (epsilon) |
| PITPNB | 0.741412 | 271 | phosphatidylinositol transfer protein, beta |
| BZW1 | 0.744141 | 451 | basic leucine zipper and W2 domains 1 |
| TRRAP | 0.749281 | 3830 | transformation/transcription domain-associated protein |
| RPS11 | 0.750099 | 158 | ribosomal protein S11 |
| EEF2 | 0.751683 | 858 | eukaryotic translation elongation factor 2 |
| RPS16 | 0.752835 | 146 | ribosomal protein S16 |
| RPS5 | 0.752888 | 204 | ribosomal protein S5 |
| PSMD7 | 0.75464 | 324 | proteasome (prosome, macropain) 26S subunit, non-ATPase, 7 |
| SDC1 | 1.327317 | 310 | syndecan 1 |
| BLMH | 1.339284 | 455 | bleomycin hydrolase |
| PSMB5 | 1.340389 | 263 | proteasome (prosome, macropain) subunit, beta type, 5 |
| PSMA2 | 1.341406 | 234 | proteasome (prosome, macropain) subunit, alpha type, 2 |
| RPS14 | 1.3489 | 151 | ribosomal protein S14 |
| ARL8B | 1.349007 | 186 | ADP-ribosylation factor-like 8B |
| DIP2B | 1.35473 | 1576 | DIP2 disco-interacting protein 2 homolog B |
| VAMP3 | 1.356224 | 100 | vesicle-associated membrane protein 3 |
| SF3B3 | 1.363129 | 1217 | splicing factor 3b, subunit 3 |
| FTH1 | 1.37234 | 183 | ferritin, heavy polypeptide 1 |
| LAMP1 | 1.38563 | 417 | lysosomal-associated membrane protein 1 |
| SERPINE2 | 1.395799 | 409 | serpin peptidase inhibitor, clade E (nexin, plasminogen activator inhibitor type 1), member 2 |
| DSP | 1.39794 | 2871 | desmoplakin |
| NPEPPS | 1.397969 | 919 | aminopeptidase puromycin sensitive |
| HNRNPD | 1.406429 | 355 | heterogeneous nuclear ribonucleoprotein D (AU-rich element RNA binding protein 1) |
| SCARB2 | 1.407609 | 478 | scavenger receptor class B, member 2 |
| CFL1 | 1.413491 | 166 | cofilin 1 (non-muscle) |
| TFPI2 | 1.414035 | 235 | tissue factor pathway inhibitor 2 |
| PUS7 | 1.418221 | 661 | pseudouridylate synthase 7 homolog |
| TNFAIP8 | 1.421807 | 198 | tumor necrosis factor, alpha-induced protein 8 |
| GLB1 | 1.436999 | 677 | galactosidase, beta 1 |
| SERPINB12 | 1.440681 | 405 | serpin peptidase inhibitor, clade B (ovalbumin), member 12 |
| TGFBI | 1.450629 | 683 | transforming growth factor, beta-induced |
| PSMB6 | 1.453919 | 239 | proteasome (prosome, macropain) subunit, beta type, 6 |
| UBE2O | 1.470022 | 1292 | ubiquitin-conjugating enzyme E2O |
| H1F0 | 1.484791 | 194 | H1 histone family, member 0 |
| ATP5B | 1.491071 | 529 | ATP synthase, H+ transporting, mitochondrial F1 complex, beta polypeptide |
| SRSF3 | 1.494334 | 164 | serine/arginine-rich splicing factor 3 |
| GNB1 | 1.494595 | 340 | guanine nucleotide binding protein (G protein), beta polypeptide 1 |
| ADAMTS1 | 1.504498 | 967 | ADAM metallopeptidase with thrombospondin type 1 motif, 1 |
| ANXA8L1 | 1.531262 | 327 | annexin A8-like 1 |
| LTF | 1.539988 | 710 | lactotransferrin |
| KPRP | 1.543578 | 579 | keratinocyte proline-rich protein |
| RAB35 | 1.544182 | 201 | RAB35, member RAS oncogene family |
| DSC1 | 1.551785 | 894 | desmocollin 1 |
| TMCO1 | 1.566566 | 188 | transmembrane and coiled-coil domains 1 |
| HNRNPU | 1.584971 | 825 | heterogeneous nuclear ribonucleoprotein U (scaffold attachment factor A) |
| NAA50 | 1.589871 | 169 | N(alpha)-acetyltransferase 50, NatE catalytic subunit |
| NACA | 1.595833 | 925 | nascent polypeptide-associated complex |
|  |  |  | alpha subunit |
| PTRF | 1.606905 | 390 | polymerase I and transcript release factor |
| PACSIN3 | 1.610612 | 424 | protein kinase C and casein kinase substrate in neurons 3 |
| NOV | 1.614804 | 357 | nephroblastoma overexpressed |
| DSG1 | 1.646823 | 1049 | desmoglein 1 |
| CPZ | 1.67634 | 652 | carboxypeptidase Z |
| PIP | 1.706199 | 146 | prolactin-induced protein |
| STK10 | 1.709256 | 968 | serine/threonine kinase 10 |
| CSRP1 | 1.713118 | 193 | cysteine and glycine-rich protein 1 |
| MDK | 1.721235 | 143 | midkine (neurite growth-promoting factor 2) |
| CSTA | 1.734742 | 98 | cystatin A (stefin A) |
| HBB | 1.750813 | 147 | hemoglobin, beta |
| SFRP1 | 1.758416 | 314 | secreted frizzled-related protein 1 |
| RPL36A | 1.777399 | 142 | ribosomal protein L36a |
| SPTAN1 | 1.784034 | 2477 | spectrin, alpha, non-erythrocytic 1 |
| HEXB | 1.792949 | 556 | hexosaminidase B (beta polypeptide) |
| SERF2 | 1.812015 | 170 | small EDRK-rich factor 2 |
| CYR61 | 1.821802 | 381 | cysteine-rich, angiogenic inducer, 61 |
| TGM3 | 1.842508 | 693 | transglutaminase 3 (E polypeptide, protein-glutamine-gamma-glutamyltransferase) |
| S100A7 | 1.856711 | 101 | S100 calcium binding protein A7 |
| DCD | 1.8685 | 110 | dermcidin |
| SNRPA | 1.919596 | SNRPA | small nuclear ribonucleoprotein polypeptide A |
| CASP14 | 1.926666 | 242 | caspase 14, apoptosis-related cysteine peptidase |
| TFPI | 1.958781 | 304 | tissue factor pathway inhibitor (lipoprotein-associated coagulation inhibitor) |
| HMGB1 | 2.181756 | 215 | high mobility group box 1 |
| GDF6 | 2.319274 | 455 | growth differentiation factor 6 |
| SERPINE1 | 2.67353 | 402 | serpin peptidase inhibitor, clade E (nexin, plasminogen activator inhibitor type 1), member 1 |
| CTGF | 3.112405 | 349 | connective tissue growth factor |
| HTRA1 | 3.777745 | 480 | HtrA serine peptidase 1 |
| COL1A1 | 22.85282 | 1464 | collagen, type I, alpha 1 |
| ATP5A1; HEL-S-123m | 1.361048 | NA | NA |
| IGHG3 | 0.284967 | NA | NA |
| HEL-S-106; LAP3 | 0.740784 | NA | NA |
| SOD1; HEL-S-44 | 1.451345 | NA | NA |
| HEL-S-43; S100A11 | 1.330245 | NA | NA |
| HEL-S-68p; PGK1 | 1.330716 | NA | NA |
| FGB | 0.416058 | NA | NA |
| IGHM | 0.680808 | NA | NA |
| C3; HEL-S-62p | 0.597758 | NA | NA |
| HSPA9; HEL-S-124m | 0.75152 | NA | NA |
| DKFZp686G21220; DKFZp686L19235; IGHA1 | 0.583982 | NA | NA |
| GC; HEL-S-51 | 0.636989 | NA | NA |

**
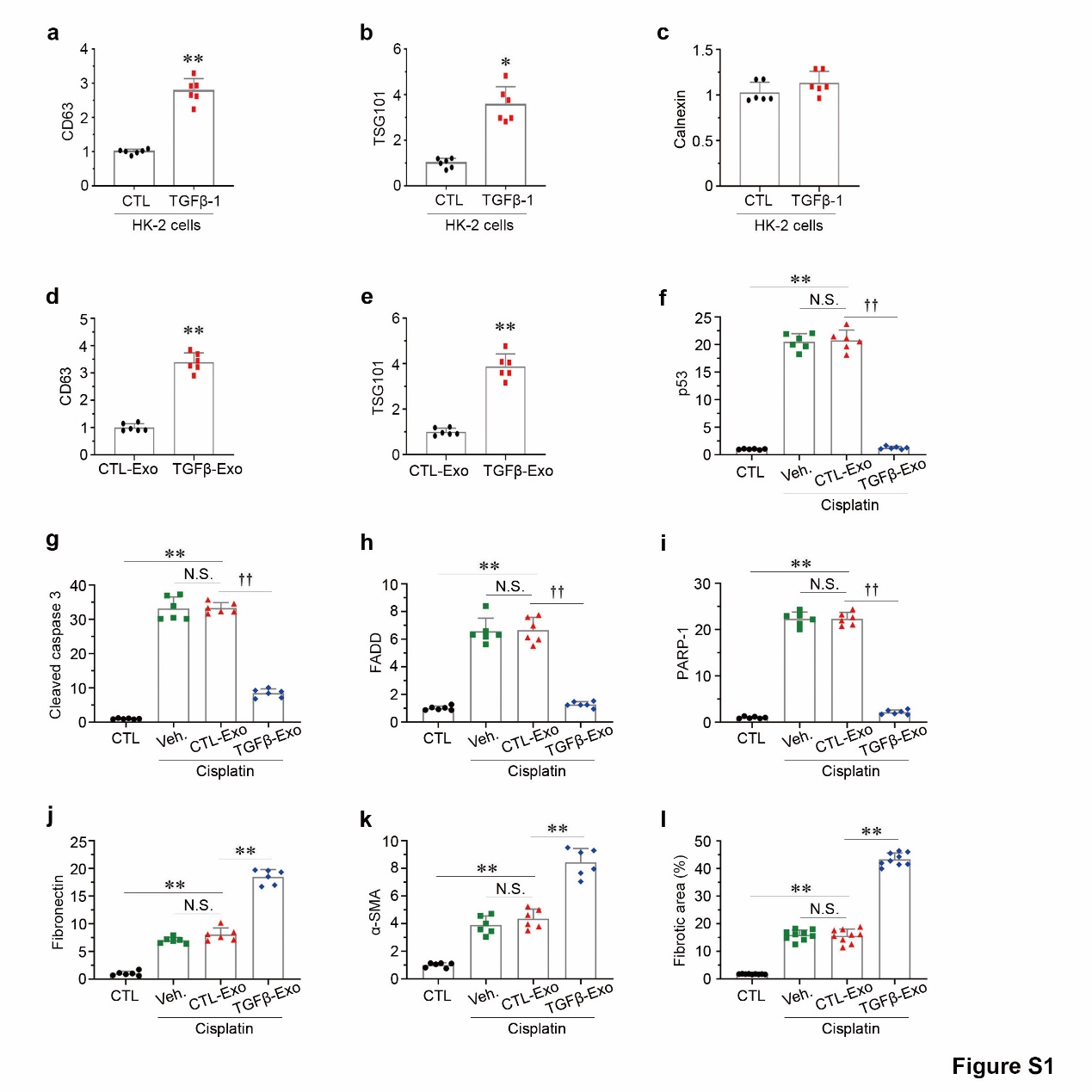
**

**
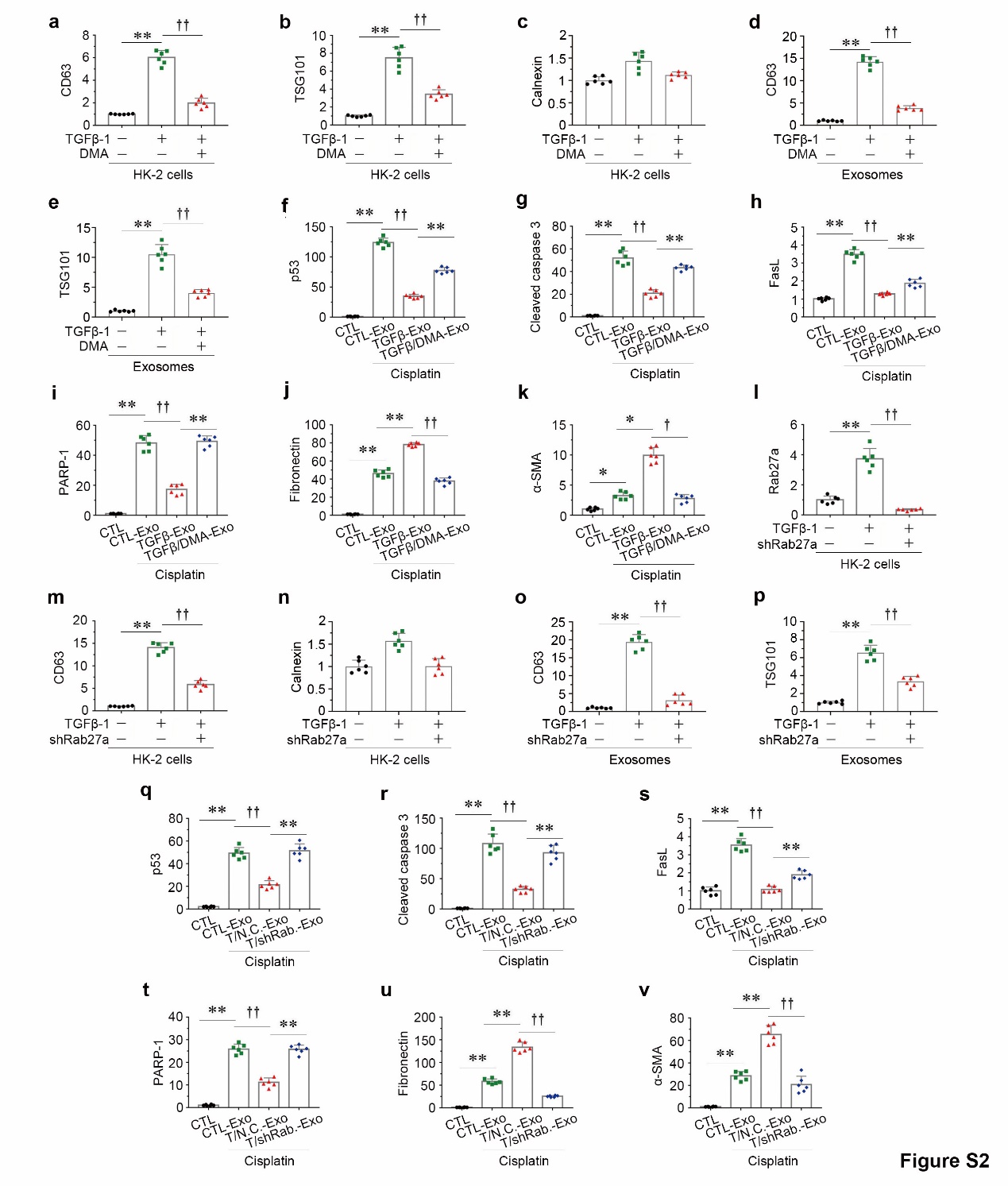
**

**
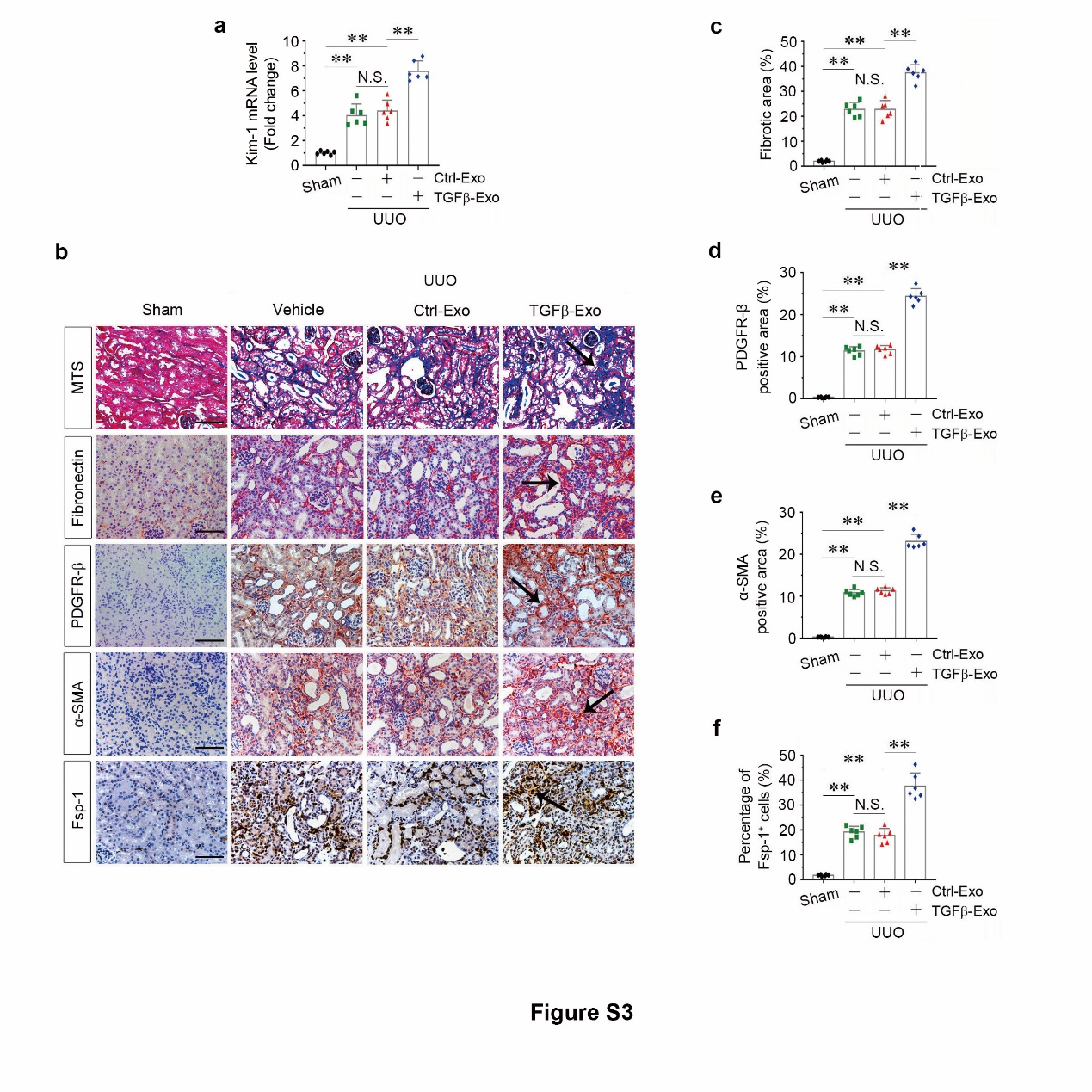
**

**
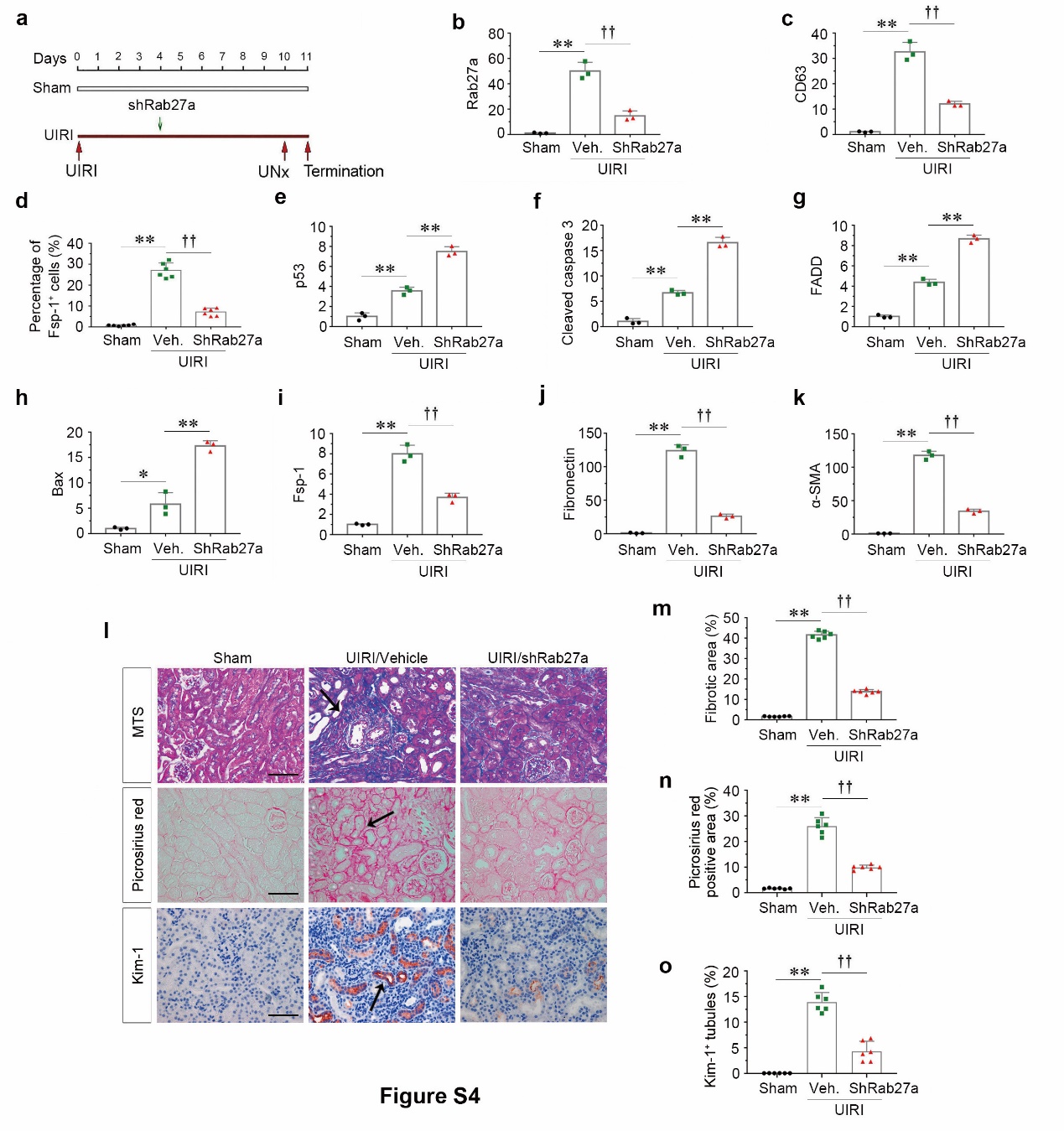
**

**
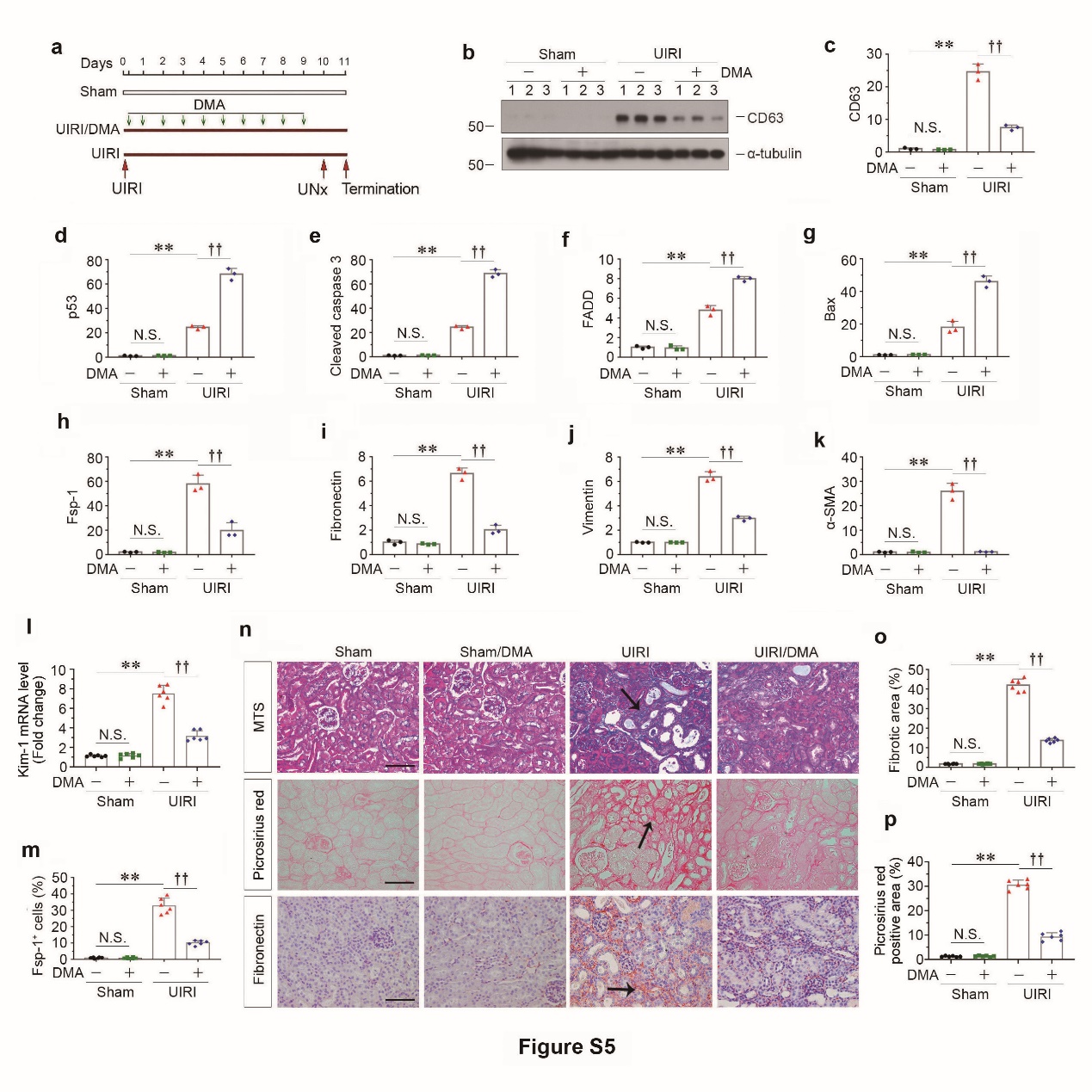
**

**
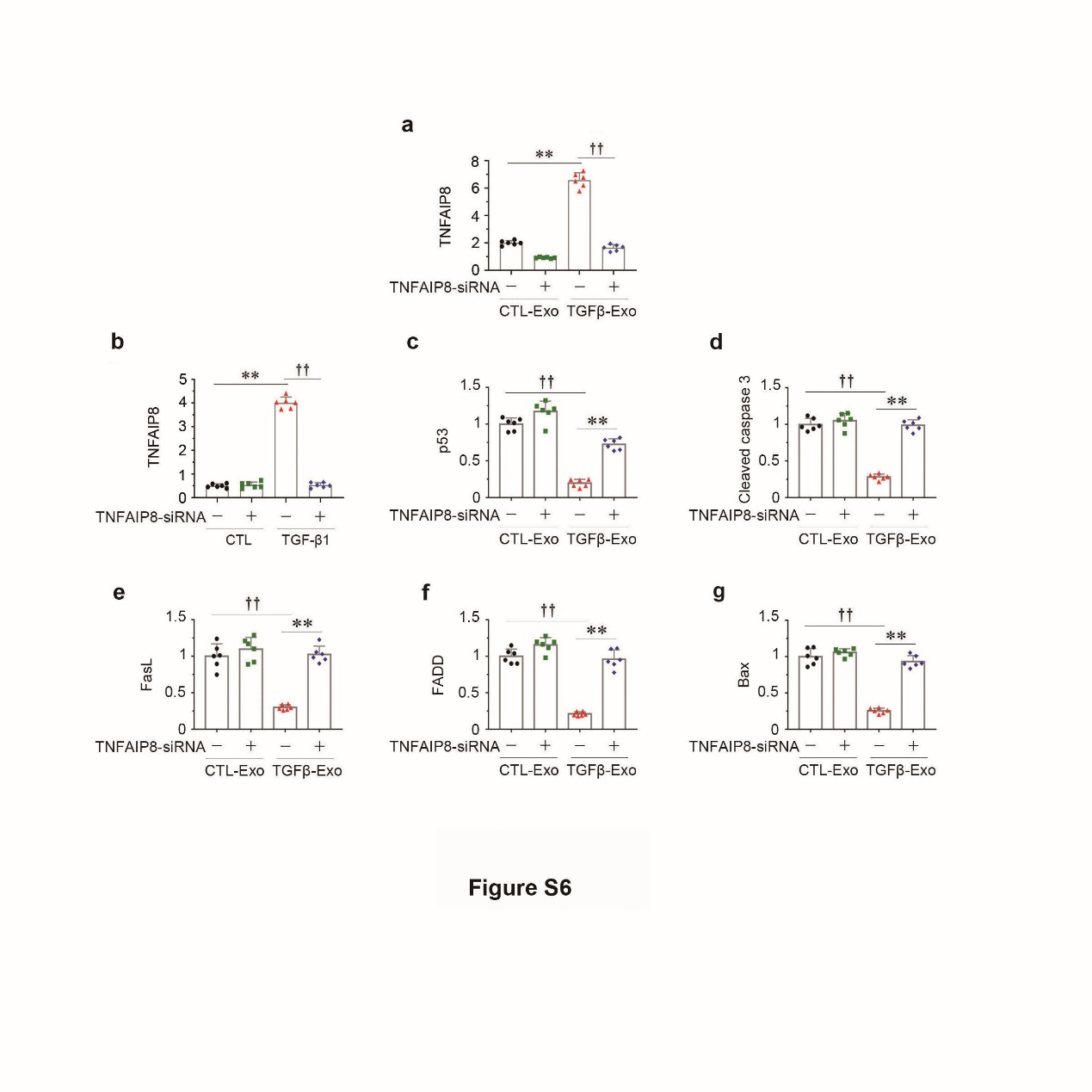
**

**
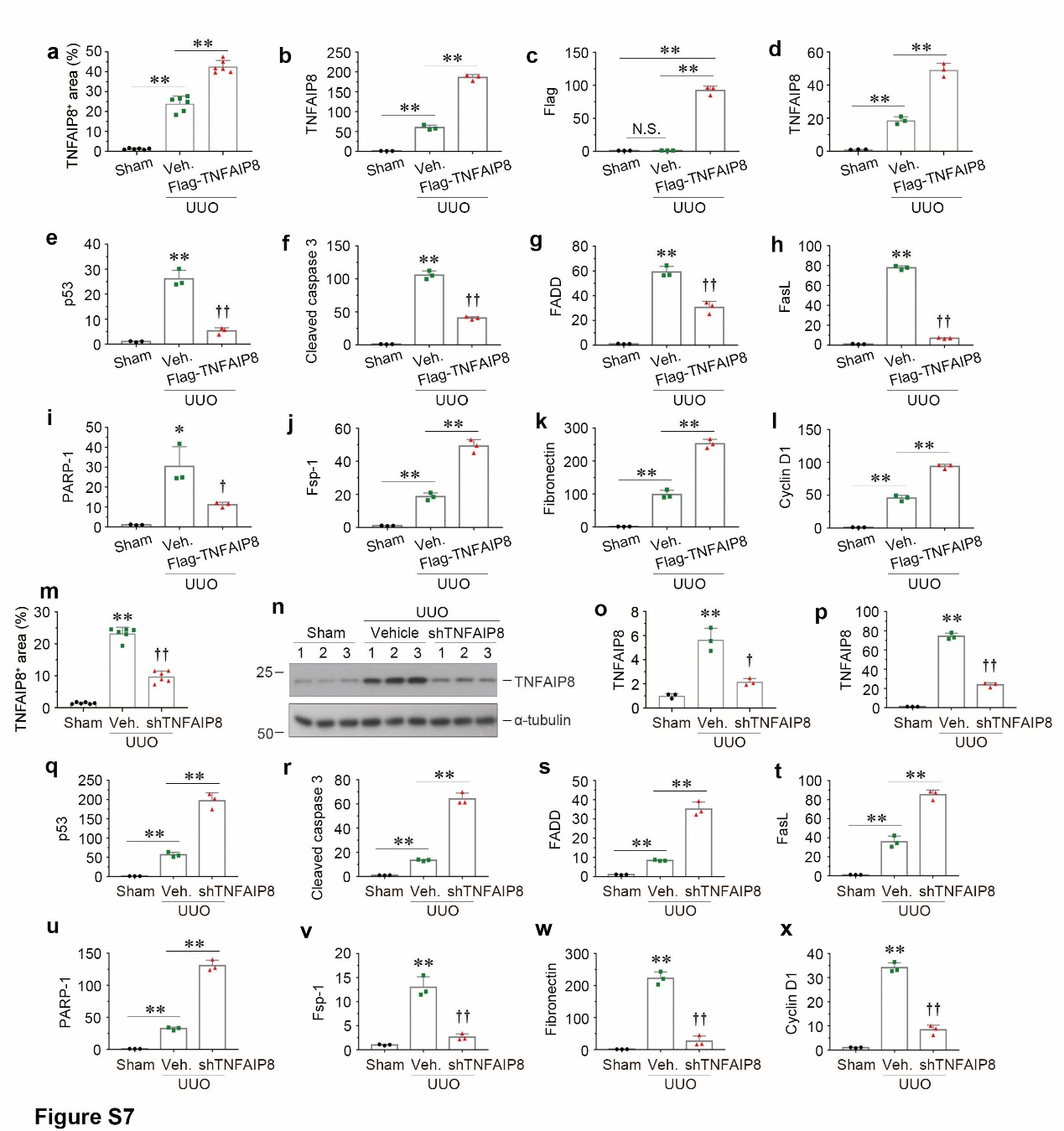
**

**
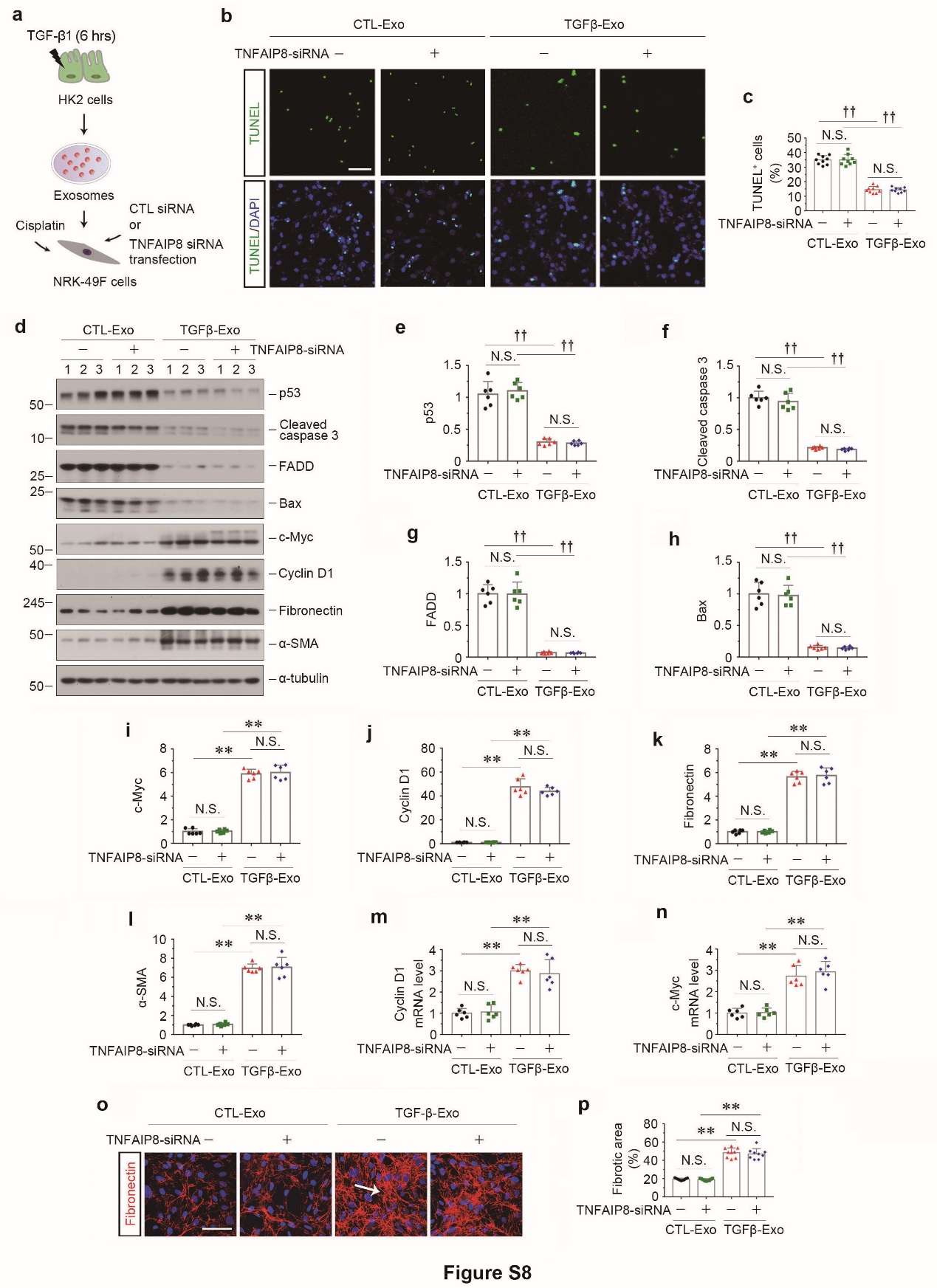
**

**
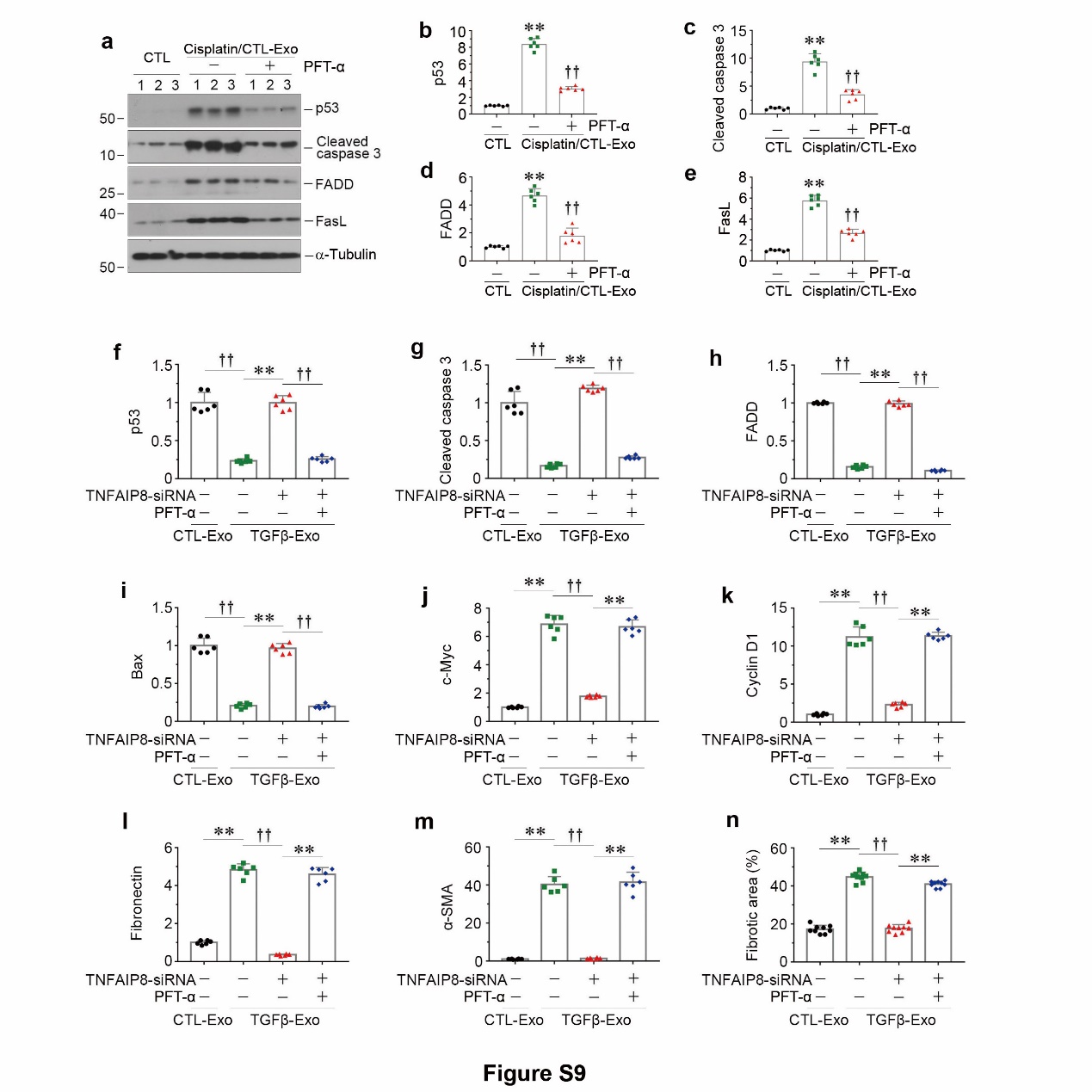
**
