# Supplementary figures and images for: Kidney tubular epithelial cells control interstitial fibroblast fate by releasing TNFAIP8-encapsulated exosomes
Source: Cell Death Dis. 2023 Oct 12;14(10):672. doi: 10.1038/s41419-023-06209-w (PMC10570316; doi:10.1038/s41419-023-06209-w)

**Supplemental Material - Original Blots**

**
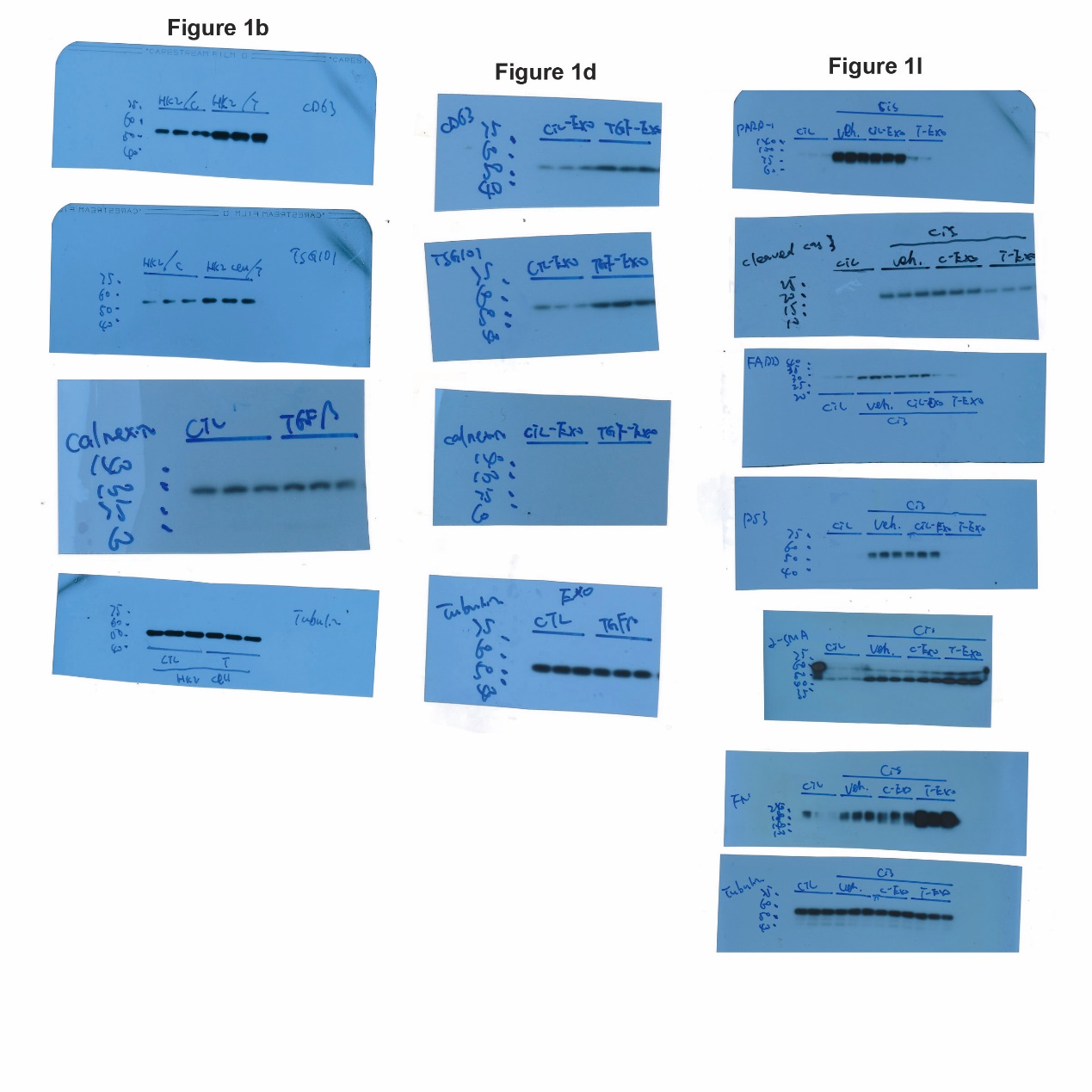
**

**
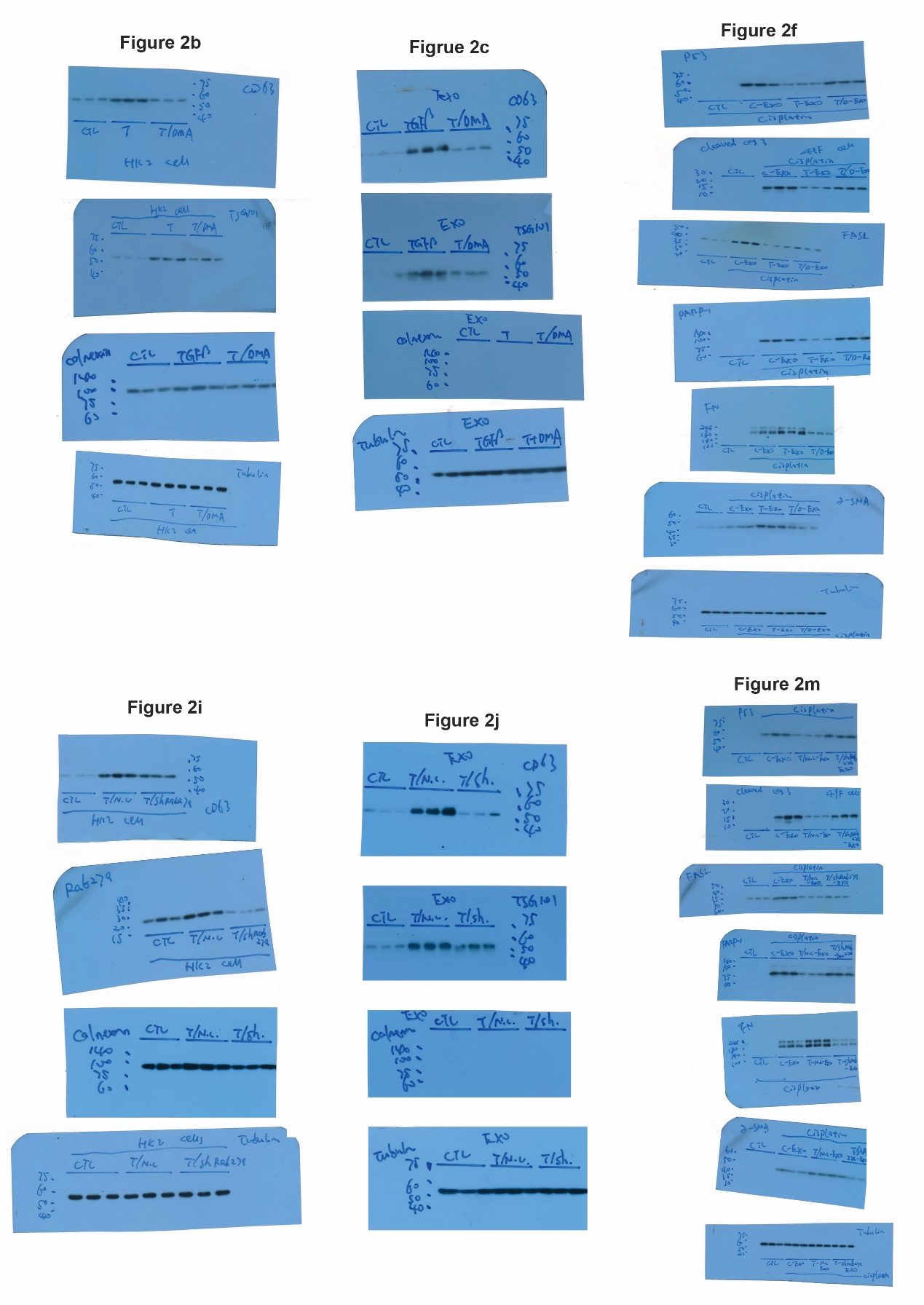
**

**
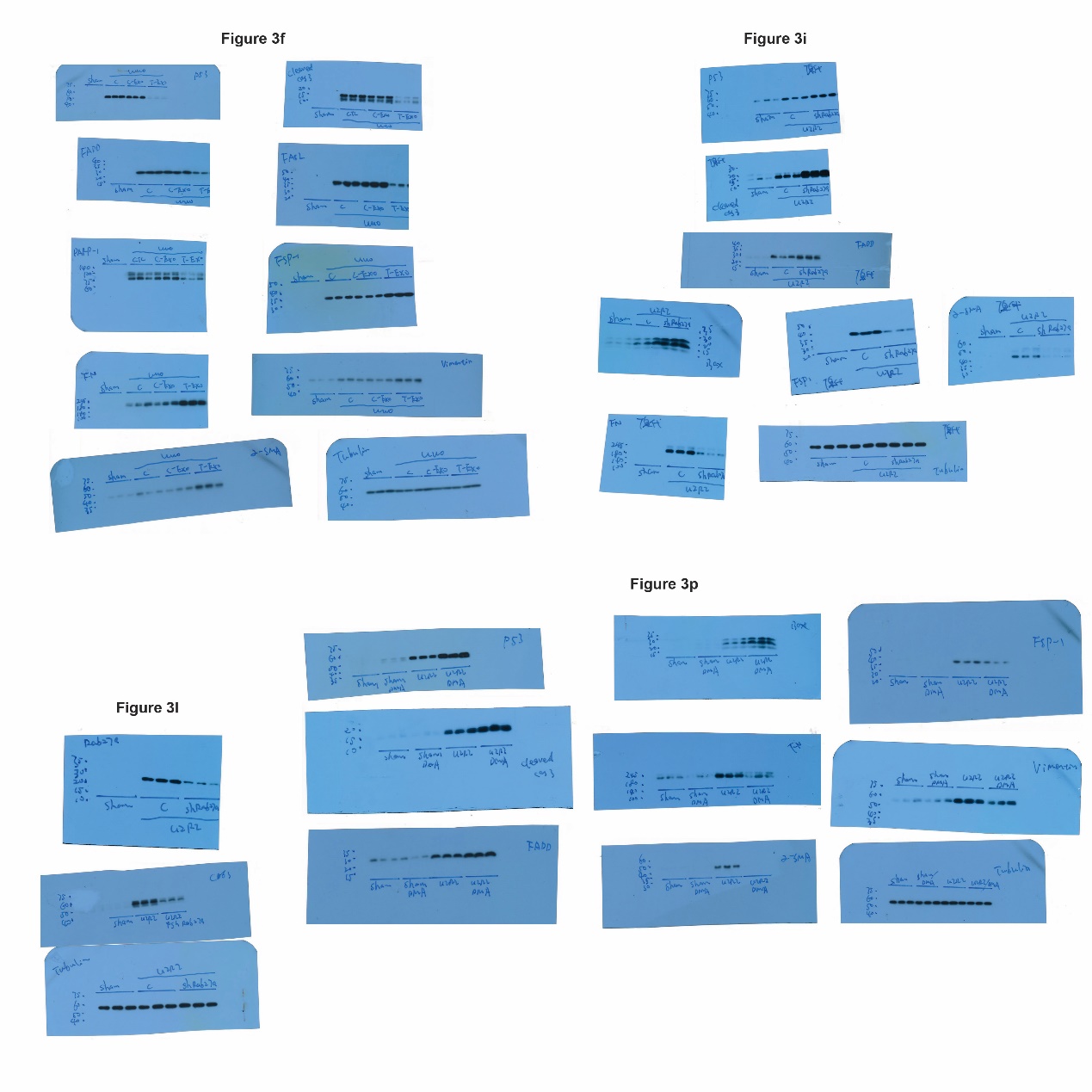
**

**
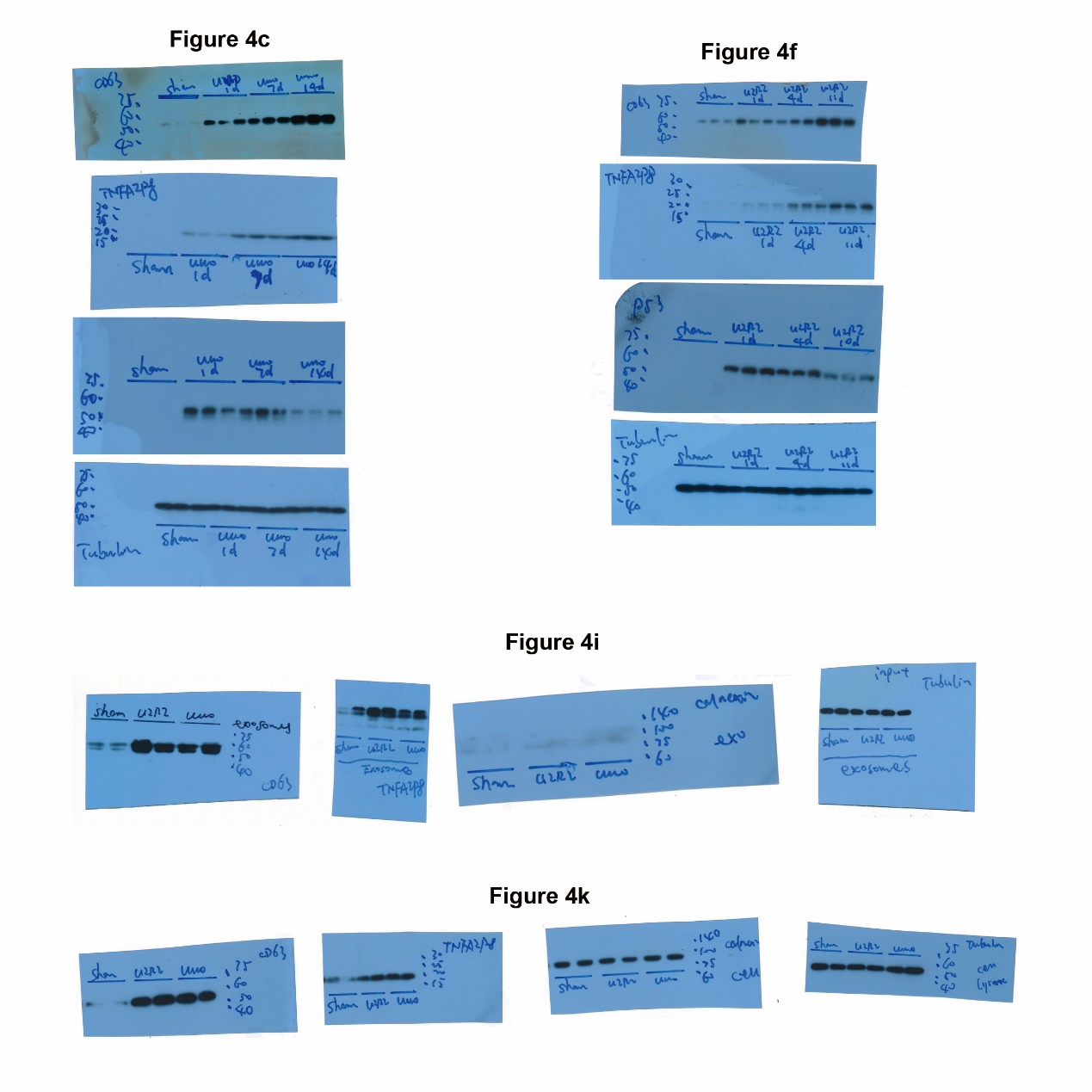
**

**
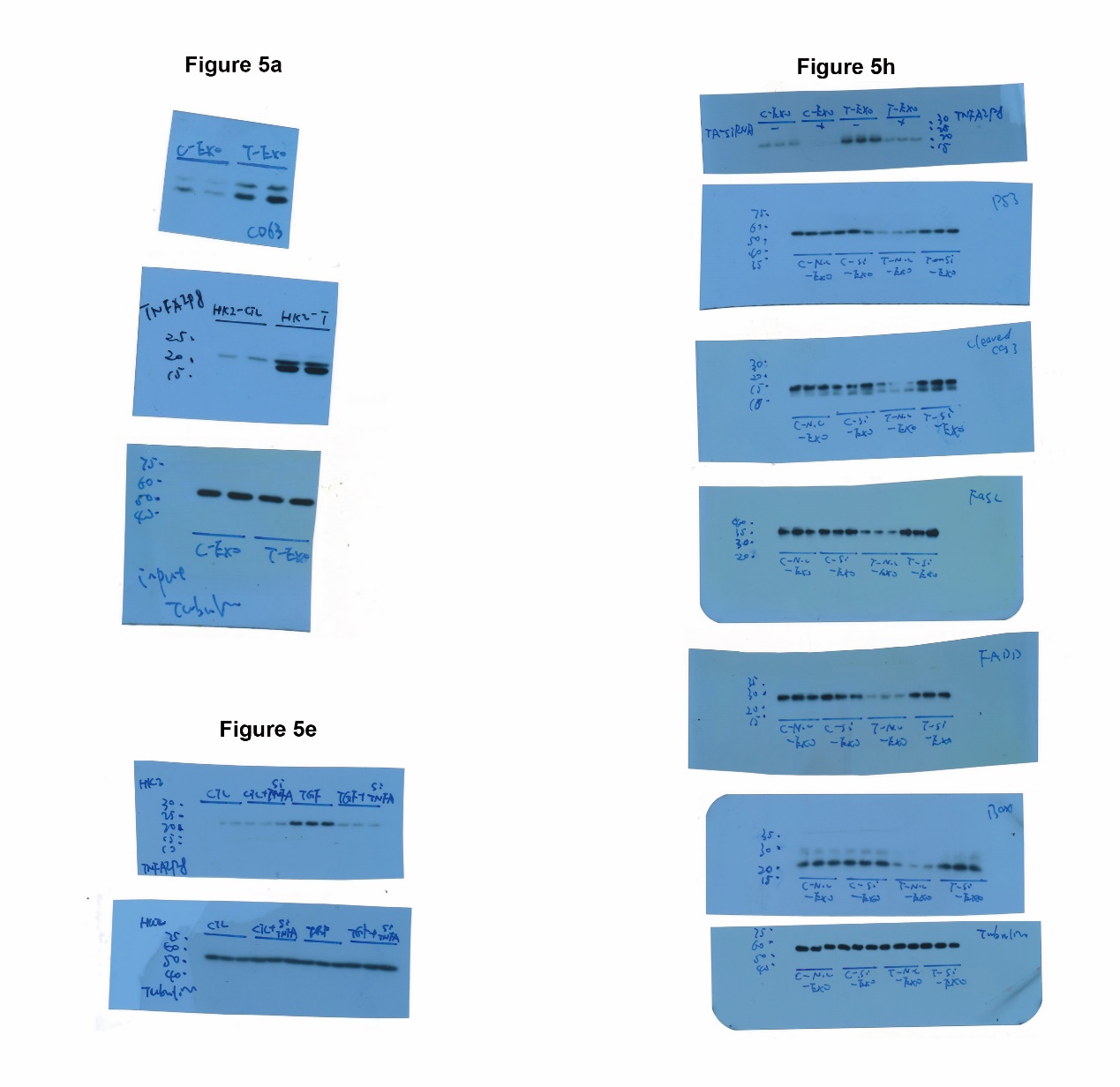
**

**
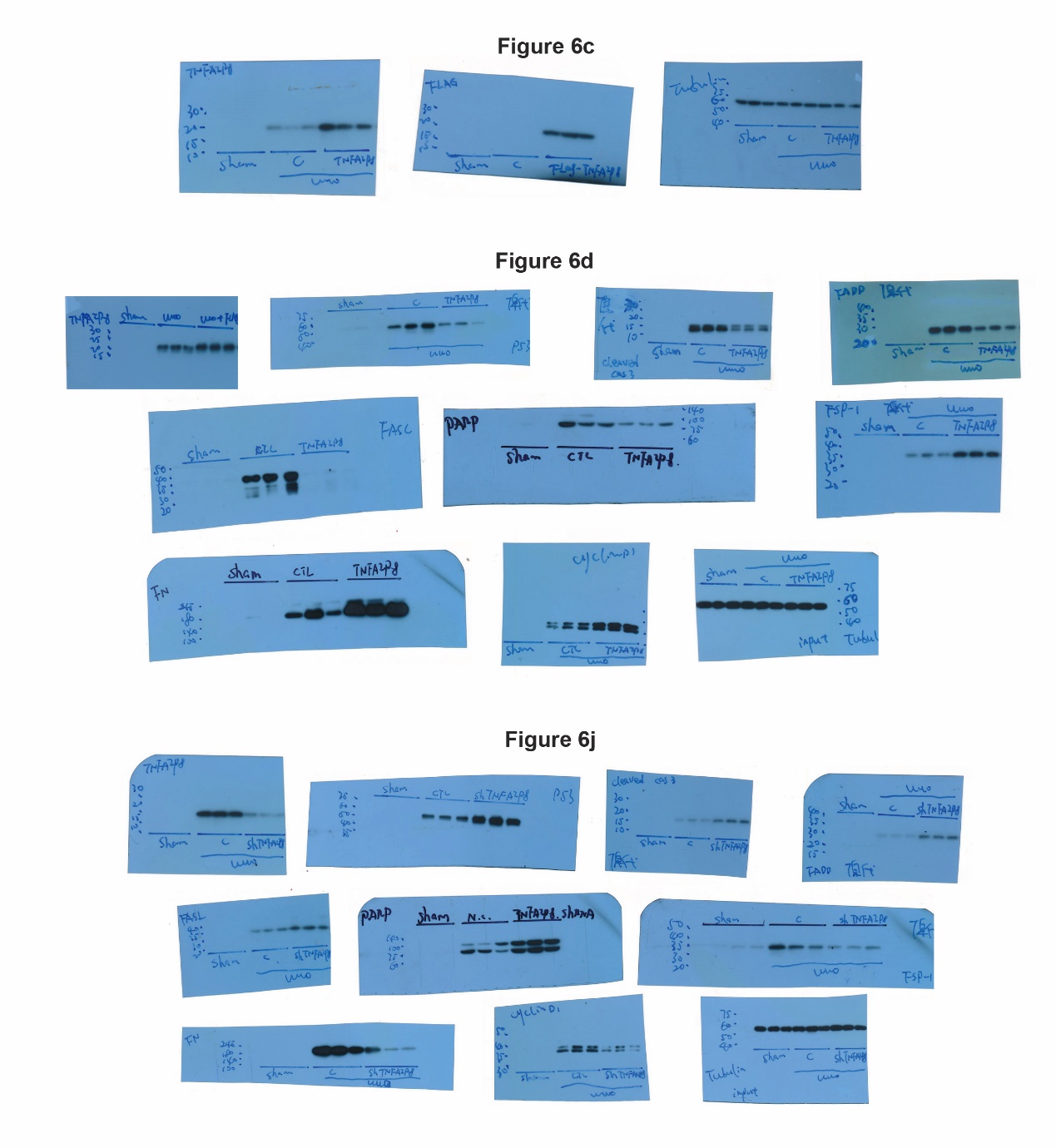
**

**
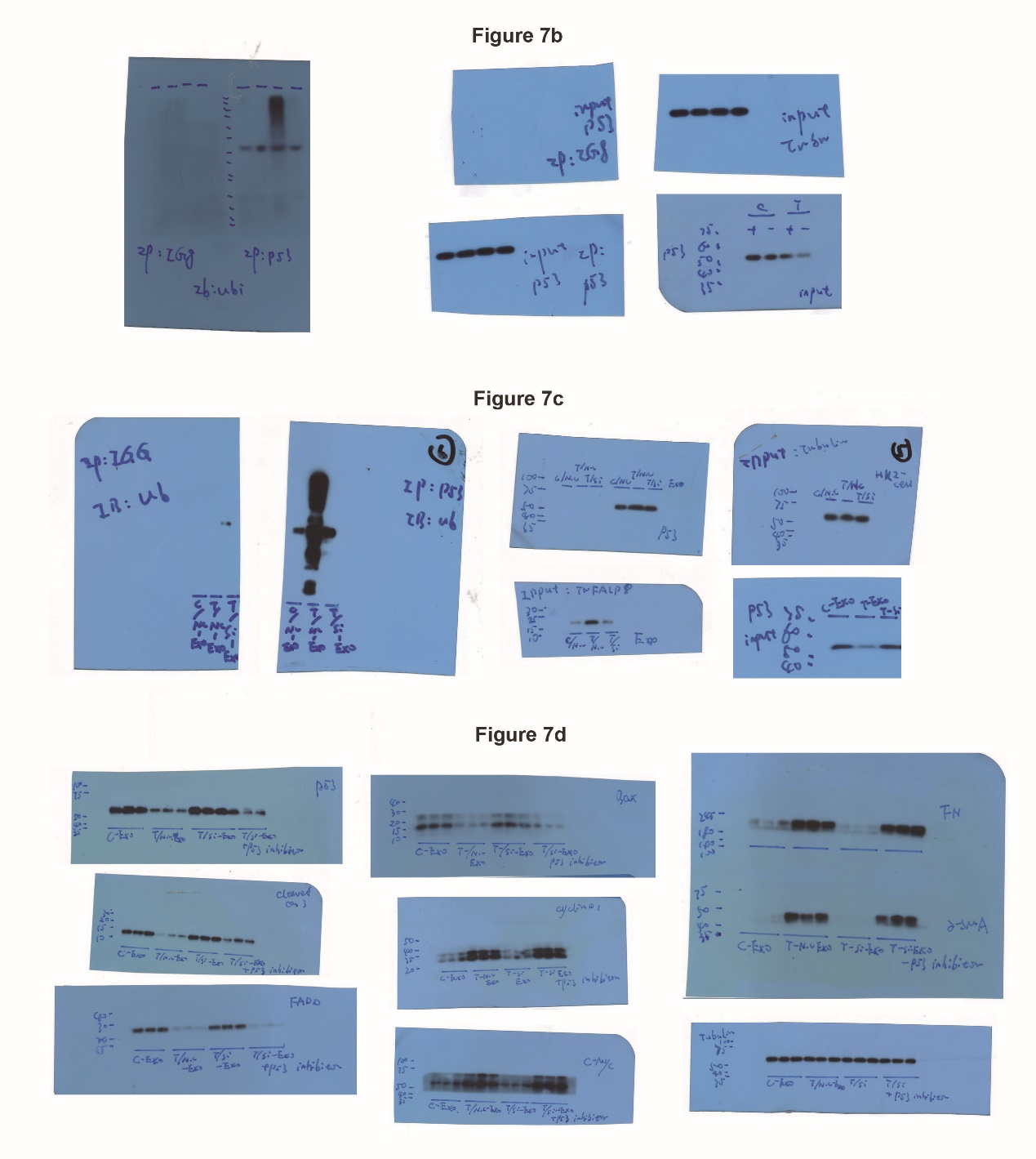
**

**
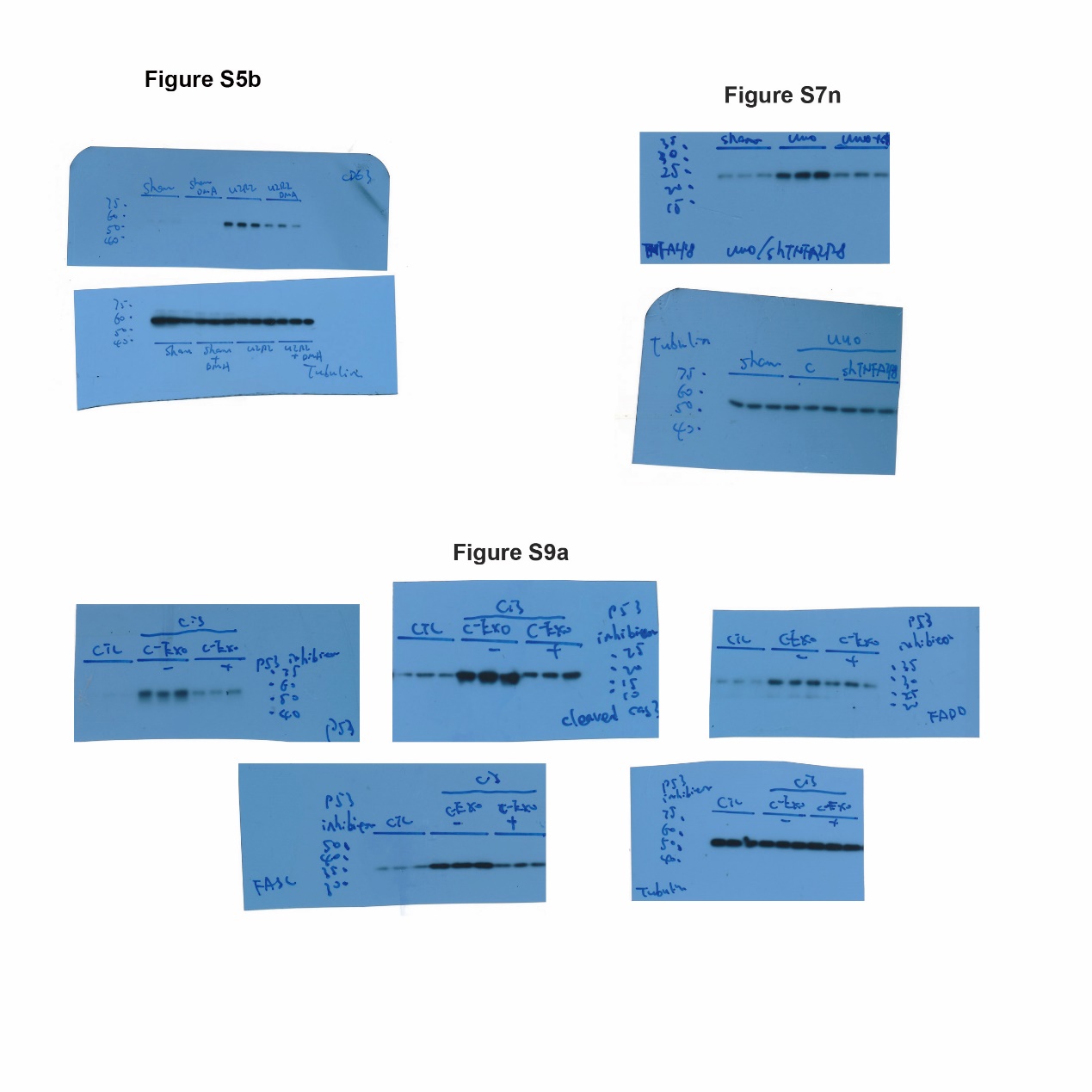
**

**
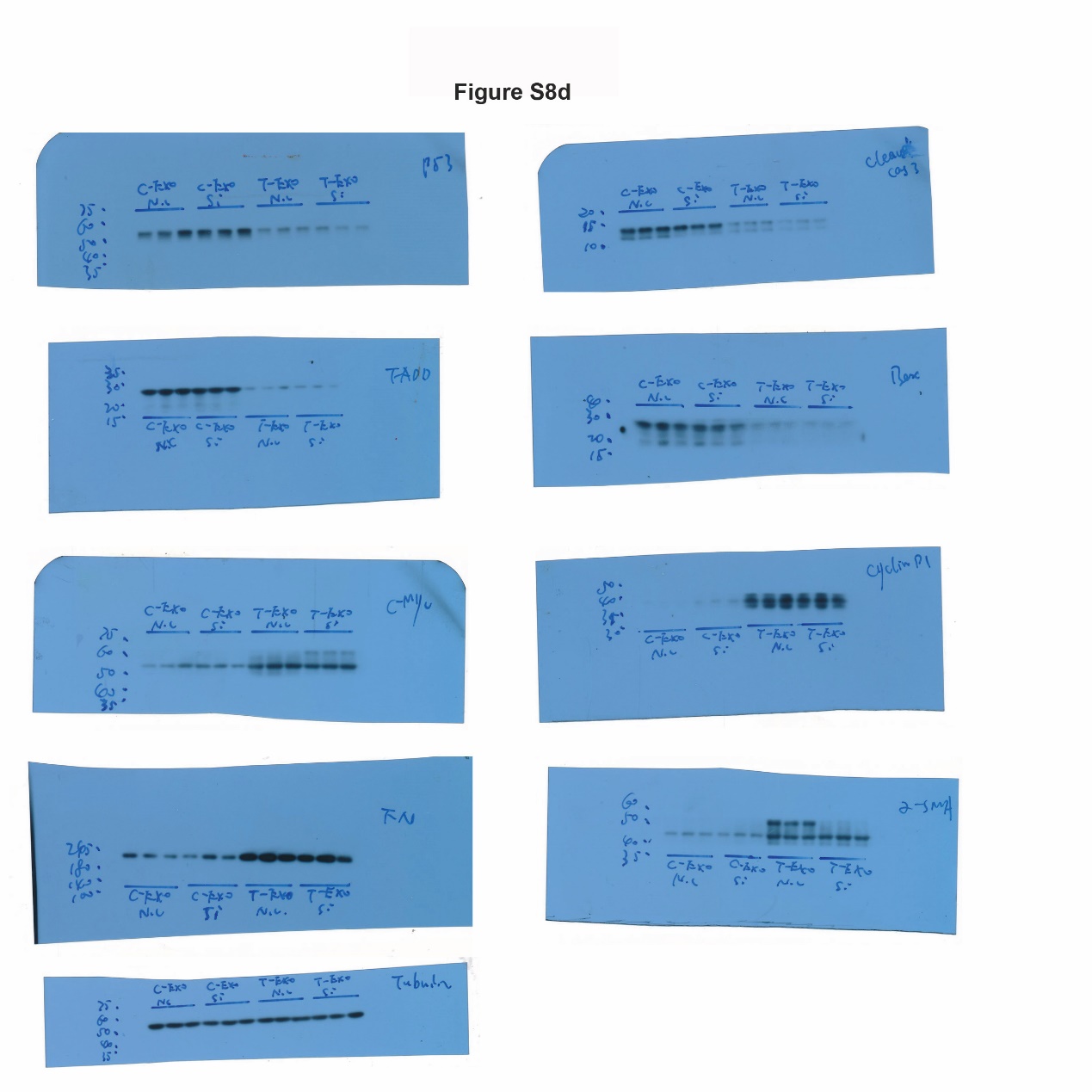
**

Supplement: Supplementary file 2 — Supplemental Material-wb [file 41419_2023_6209_MOESM2_ESM.docx]
